# Actin Re-Organization Induced by *Chlamydia trachomatis* Serovar D - Evidence for a Critical Role of the Effector Protein CT166 Targeting Rac

**DOI:** 10.1371/journal.pone.0009887

**Published:** 2010-03-25

**Authors:** Jessica Thalmann, Katrin Janik, Martin May, Kirsten Sommer, Jenny Ebeling, Fred Hofmann, Harald Genth, Andreas Klos

**Affiliations:** 1 Institute for Medical Microbiology and Hospital Epidemiology, Hannover Medical School, Hannover, Germany; 2 Institute for Toxicology, Hannover Medical School, Hannover, Germany; Technical University Munich, Germany

## Abstract

The intracellular bacterium *Chlamydia trachomatis* causes infections of urogenital tract, eyes or lungs. Alignment reveals homology of CT166, a putative effector protein of urogenital *C. trachomatis* serovars, with the N-terminal glucosyltransferase domain of clostridial glucosylating toxins (CGTs). CGTs contain an essential DXD-motif and mono-glucosylate GTP-binding proteins of the Rho/Ras families, the master regulators of the actin cytoskeleton. CT166 is preformed in elementary bodies of *C. trachomatis* D and is detected in the host-cell shortly after infection. Infection with high MOI of *C. trachomatis* serovar D containing the *CT166* ORF induces actin re-organization resulting in cell rounding and a decreased cell diameter. A comparable phenotype was observed in HeLa cells treated with the Rho-GTPase-glucosylating Toxin B from *Clostridium difficile* (TcdB) or HeLa cells ectopically expressing CT166. CT166 with a mutated DXD-motif (CT166-mut) exhibited almost unchanged actin dynamics, suggesting that CT166-induced actin re-organization depends on the glucosyltransferase motif of CT166. The cytotoxic necrotizing factor 1 (CNF1) from *E. coli* deamidates and thereby activates Rho-GTPases and transiently protects them against TcdB-induced glucosylation. CNF1-treated cells were found to be protected from TcdB- and CT166-induced actin re-organization. CNF1 treatment as well as ectopic expression of non-glucosylable Rac1-G12V, but not RhoA-G14A, reverted CT166-induced actin re-organization, suggesting that CT166-induced actin re-organization depends on the glucosylation of Rac1. In accordance, over-expression of CT166-mut diminished TcdB induced cell rounding, suggesting shared substrates. Cell rounding induced by high MOI infection with *C. trachomatis* D was reduced in cells expressing CT166-mut or Rac1-G12V, and in CNF1 treated cells. These observations indicate that the cytopathic effect of *C. trachomatis* D is mediated by CT166 induced Rac1 glucosylation. Finally, chlamydial uptake was impaired in CT166 over-expressing cells. Our data strongly suggest CT166's participation as an effector protein during host-cell entry, ensuring a balanced uptake into host-cells by interfering with Rac-dependent cytoskeletal changes.

## Introduction

*Chlamydia trachomatis* is a gram negative, obligate intracellular bacterium. It causes infections of the eyes, the urogenital tract, or the lungs of newborns. Infections with the serovars D–K range from acute to chronic inflammatory diseases of the urogenital tract with sequelae such as infertility or reactive arthritis. The serovars L1–L3 cause *Lymphogranuloma venereum*, a more severe sexually transmitted urogenital infection that also affects the inguinal lymph nodes. Serovars A–C lead to trachoma, the main cause of preventable blindness worldwide.

*Chlamydiae* share a unique developmental cycle: A metabolically inactive, infectious form called the elementary body (EB) enters the host-cell. In a host-derived inclusion it differentiates into its metabolically active form called the reticulate body that multiplies by binary fission. Approximately 20 h post infection (p.i.), the reticulate bodies start to differentiate back into a new generation of infectious EBs.

The “plasticity zone” [1] in the genome of the serovars D–K, and of *C. muridarum* (a pathogen of mice) and *C. caviae* (a pathogen of guinea pig) contains an open reading frame (ORF) with sequence similarities at the amino acid level to bacterial and mammalian glucosyltransferases [2], [3]. Protein database alignment revealed homology of the N-terminal glucosyltransferase domain of clostridial glucosylating toxins (CGTs) to *CT166* [4], the 1917 bp ORF of *C. trachomatis* serovar D [5]. The essential structural element for the glucosyltransferase activity in the clostridial enzymes is a motif containing the amino acid sequence DXD, which is involved in Mn^2+^-dependent binding of UDP-glucose in the catalytic cleft [3]. Mutational exchange of both aspartic acid residues into alanines strongly reduces enzymatic activity [3], [6].

The CGT glucosyltransferases mono-glucosylate specifically low molecular weight GTP-binding proteins of the Rho/Ras families [7], [8]. The Rho proteins are master regulators of the actin cytoskeleton that cycle between a GTP-bound active conformation and a GDP-bound inactive conformation [9]. CGT-catalyzed glucosylation prevents activation of Rho/Ras proteins, leading to inhibited effector coupling and subsequent breakdown of the actin cytoskeleton of the target cell (“cytopathic effect”).

So far, it has only been shown that the hypothetical protein CT166 is actually expressed by *C. trachomatis* serovar D [2]: The protein can be detected in elementary bodies. Additionally, during the first 60 min p.i. it is present in lysates of epithelial HeLa cells that were incubated with high multiplicities of infection (MOIs) of serovar D. Infection with *C. trachomatis* D (but not L2 lacking the corresponding ORF) leads to actin re-organization and cell rounding. These findings led to the hypothesis that CT166 is a glucosyltransferase that possibly inactivates Rho proteins and subsequently causes actin re-organization.

In this study, we analyzed the biochemical and functional potential of CT166 using HeLa cells that ectopically express this putative glucosyltransferase or the corresponding protein with mutated DXD-motif. Our data show that CT166 induces actin re-organization similar to that observed upon high MOI infection of HeLa cells with *C. trachomatis* D. Our results indicate that CT166 acts on the level of Rho-proteins and suggest that glucosylation of Rac1 by CT166 might be the underlying mechanism leading to actin re-organization.

## Results

### Actin Re-organization Induced by CT166-containing *C. trachomatis* Serovar D Resembles That Induced by *C. difficile* Toxin B

**Figure 1:**
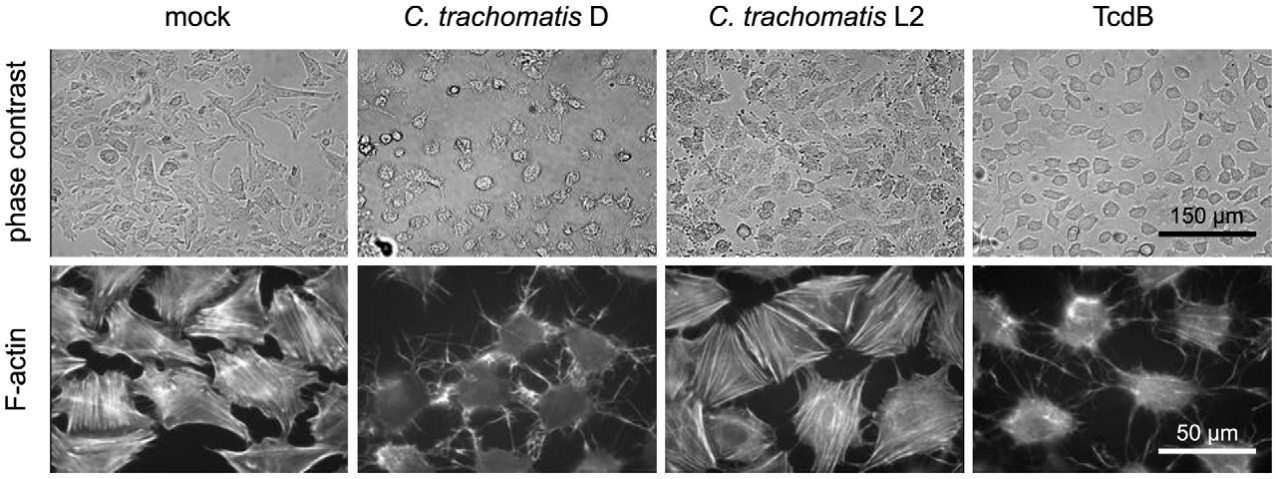
*C. trachomatis* serovar D causes actin re-organization and cell rounding comparable to Toxin B from *C. difficile* (TcdB), while the CT166 ORF lacking *C. trachomatis* serovar L2 does not. HeLa cells were mock treated, or treated with *C. trachomatis* D, *C. trachomatis* L2 (MOI 100) or TcdB (1 ng/ml for 2 h). Cells were fixed 4 h post infection and the actin cytoskeleton was stained with rhodamine-phalloidin.

*C. trachomatis* D exhibits the *CT166* ORF, a putative glucosyltransferase. To corroborate the hypothesis that CT166 induces actin re-organization HeLa cells were infected with *C. trachomatis* D and analyzed for actin re-organization. Infection with the CT166-proficient *C. trachomatis* D caused a loss of actin stress fibers and a loss of cell shape (cell rounding), as analyzed using phase contrast microscopy and fluorescence microscopy of rhodamine-phalloidin-stained cells (Figure 1). Comparable morphological changes were observed upon treatment of HeLa cells with the Rho-glucosylating *C. difficile* Toxin B (TcdB) (Figure 1). Infection of HeLa cells with the *C. trachomatis* L2 that lacks the *CT166* ORF did not cause actin re-organization of HeLa cells (Figure 1), suggesting that *C. trachomatis* D-induced actin re-organization depends on CT166.

### Expression of CT166 in *E. coli* and Generation of Specific Antiserum

Following the hypothesis that CT166 is a glucosyltransferase, CT166 and a corresponding DXD-mutant CT166-D415A.D417A (CT166-mut) were expressed as GST fusion proteins in *E. coli*, an approach that has been successfully applied for the characterization of the catalytic properties of TcdB and other glucosylating toxins [3], [4], [10]. Recombinant CT166 was analyzed for glucosyltransferase activity and for glucohydrolase activity in the presence of several [^14^C]-labeled UDP-hexoses including UDP-glucose, UDP-galactose, and UDP-N-acetylglucosamin. Unfortunately, CT166 expressed by *E. coli* exhibited neither glucosyltransferase nor glucohydrolase activity (data not shown). Affinity purified CT166-mut, however, was applied as antigen to raise anti-CT166 antiserum in rabbits (see Figure S1A and B).

### Generation of CT166 Expressing HeLa Clones

In order to generate a tool to analyze CT166 functions, CT166-wt and CT166-mut were expressed in HeLa cells, using a tetracycline-inducible expression system (TREx™ System, Invitrogen). Several HeLa clones were analyzed for expression of CT166-wt and CT166-mut using Western blot analysis with anti-CT166 antiserum (Figure S1A). Upon treatment with 1 µg/ml tetracycline all analyzed HeLa clones expressed either CT166-wt or CT166-mut. These myc-tagged proteins exhibited an apparent molecular mass of approximately 75 kDa, in good accordance with the calculated molecular mass of ∼77 kDa.

Figure S1A exemplarily presents the expression in two sets of HeLa clones, each containing one CT166-wt or CT166-mut expressing clone and a corresponding control: HeLa-CT166-wt_JT30, HeLa-CT166-mut_JT4N, and HeLa-control_JT3 (set 1), and HeLa-CT166-wt_JT26, HeLa-CT166-mut_JTB4, and HeLa-control_JT1 (set 2). Within each set the cellular concentrations of CT166-wt and CT166-mut were in a similar range, as normalized to total protein concentration or β-actin concentration. The HeLa clones from set 1 were used for all following experiments, if not indicated otherwise. A fivefold increased tetracycline concentration did not further augment the cellular CT166 concentration (Figure S1B). Some CT166 expression, however, was also observed in the absence of tetracycline (Figure S1B) showing that CT166 expression was thus not completely repressed. CT166 expression was further analyzed by immuno-cytochemistry using anti-myc antibody. HeLa-CT166-wt and HeLa-CT166-mut cells (upon tetracycline treatment) stained positive for the myc-tagged proteins in the cytosol (Figure S2). HeLa-control cells (that lacked CT166 expression) showed a slight positive staining that predominantly located to the nucleus, most likely resulting from endogenous myc (Figure S2).

**Figure 2:**
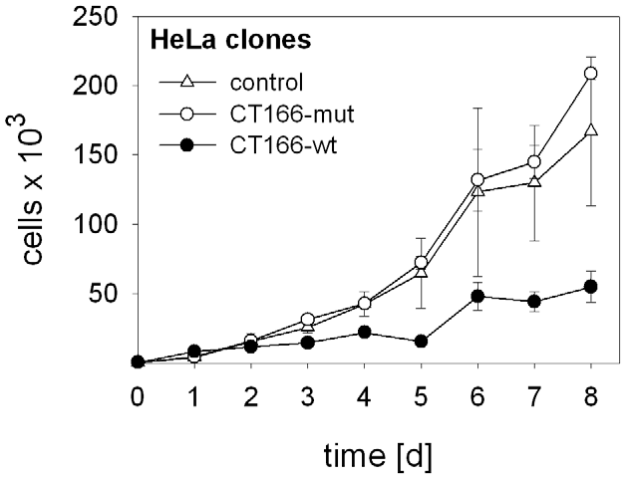
Expression of CT166 reduces proliferation rate of HeLa cells. Proliferation of HeLa-CT166-wt and HeLa-CT166-mut and non-expressing control cells, in the presence of tetracycline, was monitored by fluorescence staining of the DNA with Hoechst 33258 as described in “Material and Methods”. The CT166-mutant expressing cells (○) show a growth similar to non-expressing cells (Δ). The growth of CT166-wildtype expressing cells (•) is considerably diminished. The mean ± SD of three independent experiments is depicted.

HeLa-CT166-wt cells exhibited a reduced proliferation rate compared to HeLa-control or HeLa-CT166-mut cells (Figure 2), as analyzed in the presence of tetracycline. This finding suggested that CT166-wt expression interfered with cell cycle progression. In contrast, HeLa-CT166-mut exhibited a doubling time comparable to HeLa-control cells (Figure 2). The finding of a reduced proliferation rate suggests a critical role of the DXD-motif in the CT166 activity on cell proliferation.

### Critical Role of the DXD-motif in CT166-induced Actin Re-organization of HeLa Cells

**Figure 3:**
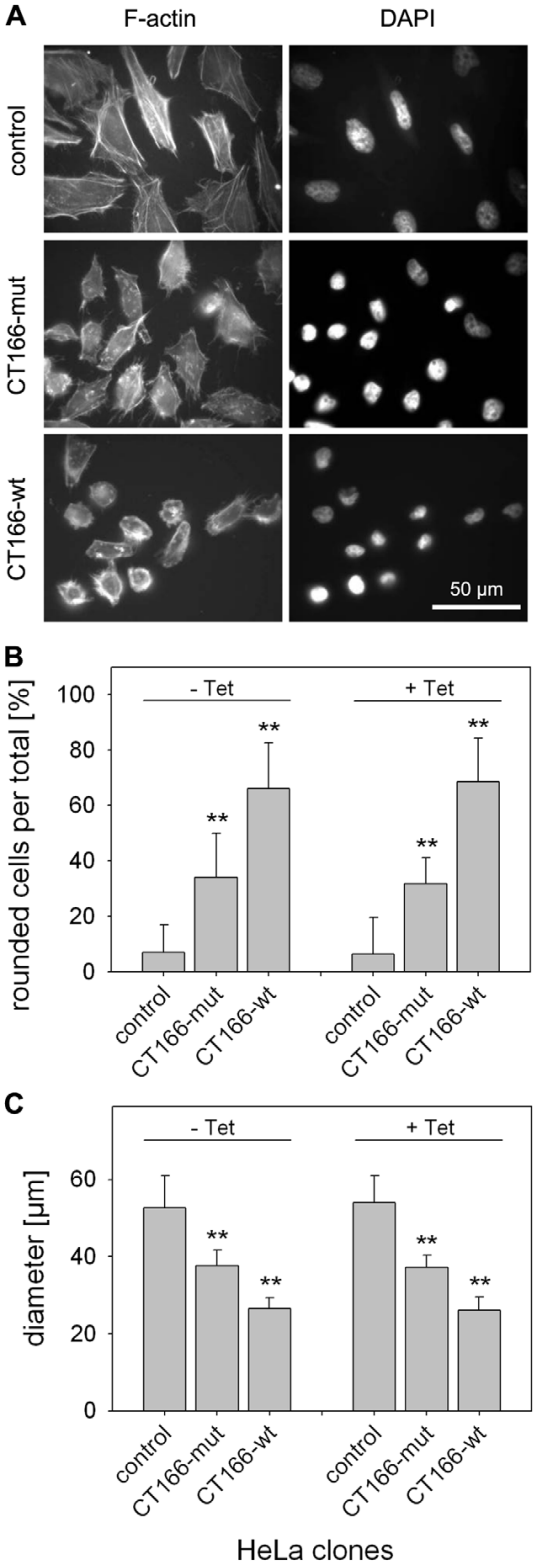
Cytopathic effect in HeLa cells stably expressing CT166 (but no multi-nucleation). HeLa-CT166-wt, HeLa-CT166-mut and HeLa-control cells were stained with rhodamine-phalloidin and DAPI after 24 h incubation with 1 µg/ml tetracycline. (**A**). Rounded cells (cell diameter <30 µm) were quantified by microscopy and given as ratio in percent. Comparison of untreated and tetracycline-treated HeLa clones reveals similar results (**B**). The average cell diameter was determined (**C**). Depicted is the mean ± SD of three independent experiments. (** indicates significant difference as compared to control cells, p<0.005.)

To corroborate the hypothesis that CT166 is responsible for actin re-organization induced by *C. trachomatis* D (Figure 1), the actin cytoskeleton of several HeLa-CT166-wt and HeLa-CT166-mut clones was visualized using rhodamine-conjugated phalloidin. Expression of CT166-wt resulted in the disappearance of actin stress fibers and loss of cell shape (cell rounding) in several clones of HeLa-CT166-wt (Figures 3A and S1C). This morphological outcome was comparable to that upon infection of HeLa cells with *C. trachomatis* D or comparable to treatment of HeLa cells with TcdB (Figure 1). CT166-induced actin re-organization was quantified. Cells exhibiting a cell diameter less than 30 µm were classified as “rounded” (“cytopathic effect”, Figures 3B and S1D). About 70% of all HeLa-CT166-wt cells were rounded, while only about 9% of HeLa-control cells exhibited this phenotype (Figures 3B and S1D). The increased cytopathic effect was reflected by a reduced average diameter of these cells (Figures 3C and S1E). The cytopathic effect in HeLa-CT166-wt cells was independent of tetracycline treatment (Figures 3B, 3C, S1D and S1E), suggesting that low CT166 expression in HeLa-CT166-wt cells as observed in the absence of tetracycline (Figure S1B) was already sufficient for the maximal cytopathic effect. This is consistent with the hypothesis that CT166 acts as an enzyme with high biological potential. Actin re-organization was further analyzed in several clones of HeLa-CT166-mut cells (Figure 3). All clones of these cells exhibited minor morphological changes compared to HeLa-CT166-wt cells including a partial loss of stress fibers, a less pronounced cytopathic effect and a higher (i.e. less reduced) average cell diameter (Figure 3). This observation suggests that CT166-mut is not a “dead” enzyme but still exhibits a faint catalytic activity. Such a faint catalytic activity that resulted in minor morphological changes has recently been described for the Rho/Ras-glucosylating *C. sordellii* Lethal toxin (TcsL) with mutated DXD-motif [6].

In the T-Rex expression system, CT166 and the corresponding mutant are tagged at the C-terminus. To confirm the findings obtained with the stably transfected HeLa cell lines and to further exclude clonal artifacts, we generated constructs for transient expression of N-terminal 6x-*c*-myc-tagged fusion proteins of CT166-wt and CT166-mut. Successful expression of the myc-tagged proteins was visualized by immuno-cytochemistry using anti-myc antibody (Figure S3). Transient expression of CT166-wt (but not CT166-mut) induced actin re-organization comparable to that observed in HeLa-CT166-wt cells. As expected, transient transfection resulted in distinct CT166 concentrations in each individual cell, as estimated from different fluorescence intensities (Figure S3). Regardless of these distinct cellular concentrations of CT166-wt all cells exhibited actin re-organization (cell rounding), showing that relatively low concentrations of CT166-wt were sufficient for the induction of actin re-organization. In contrast, even cells expressing high amounts of CT166-mut did not show the same rounded phenotype as CT166-wt transfected cells. These observations confirmed that CT166-wt was sufficient for actin re-organization and that the DXD-motif was critical for the cytopathic activity of CT166.

### Possible Role of the Rho-family Protein Rac1 in CT166-induced Actin Re-organization

Rho proteins are susceptible to TcdB-catalyzed glucosylation specifically in their inactive GDP-bound state. In their GTP-bound state, the acceptor amino acid (Thr-35 in Rac1, Thr-37 in RhoA) is involved in GTP coordination and therefore not accessible for glucosylation; active Rho proteins are thus protected from glucosylation [11]–[13].

**Figure 4:**
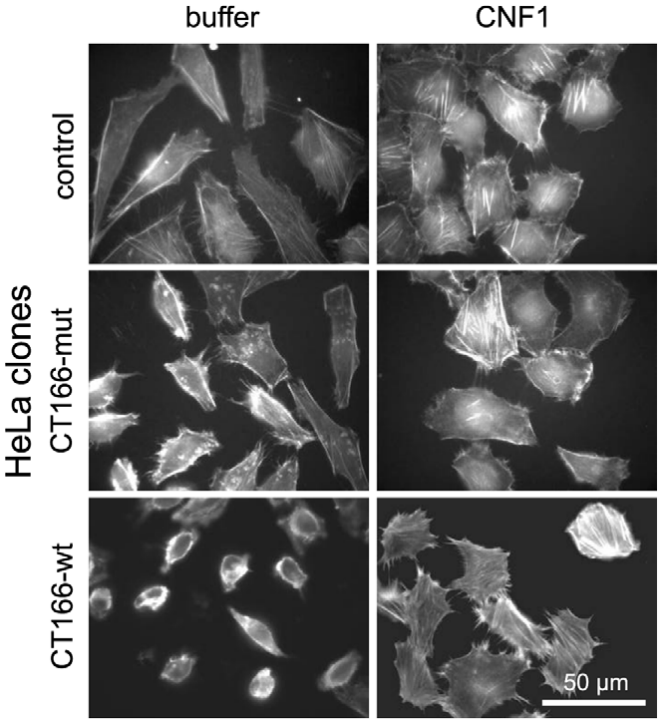
CNF1 reverts the CT166-induced phenotype. HeLa-CT166-wt, HeLa-CT166-mut and HeLa-control cells were treated with tetracycline for 24 h and subsequently treated with 0.1 µg/ml CNF1 from *E. coli* for 6 h. Actin cytoskeleton was stained with rhodamine-phalloidin. One representative experiment (n = 3) is depicted.

Based on the assumption that CT166-induced actin re-organization is mediated by glucosylation of Rho proteins, activation of Rho proteins should prevent CT166-induced actin re-organization. Rho proteins were activated using the cytotoxic necrotizing factor 1 (CNF1) from *E. coli*, that deamidates and thereby constitutively activates a broad-spectrum of Rho proteins [14], [15]. CNF1 treatment of HeLa cells prevented TcdB-induced actin re-organization in HeLa cells (Figure S4), corroborating published data [16]. CNF1 treatment of HeLa-CT166-wt cells induced stress fiber formation and rounded cells spread out (Figure 4). CNF1-induced activation of Rho proteins was thus sufficient to prevent CT166-induced actin re-organization (“protective effect”). The treatment of HeLa-CT166-wt with CNF1 did not interfere with the ectopic expression of CT166, as the cellular CT166 level was comparable in the presence or absence of CNF1 (Figure S5A), excluding the possibility that the reduction in cell rounding was based on reduced CT166 expression. These observations suggested that Rho proteins may be targets of CT166.

**Figure 5:**
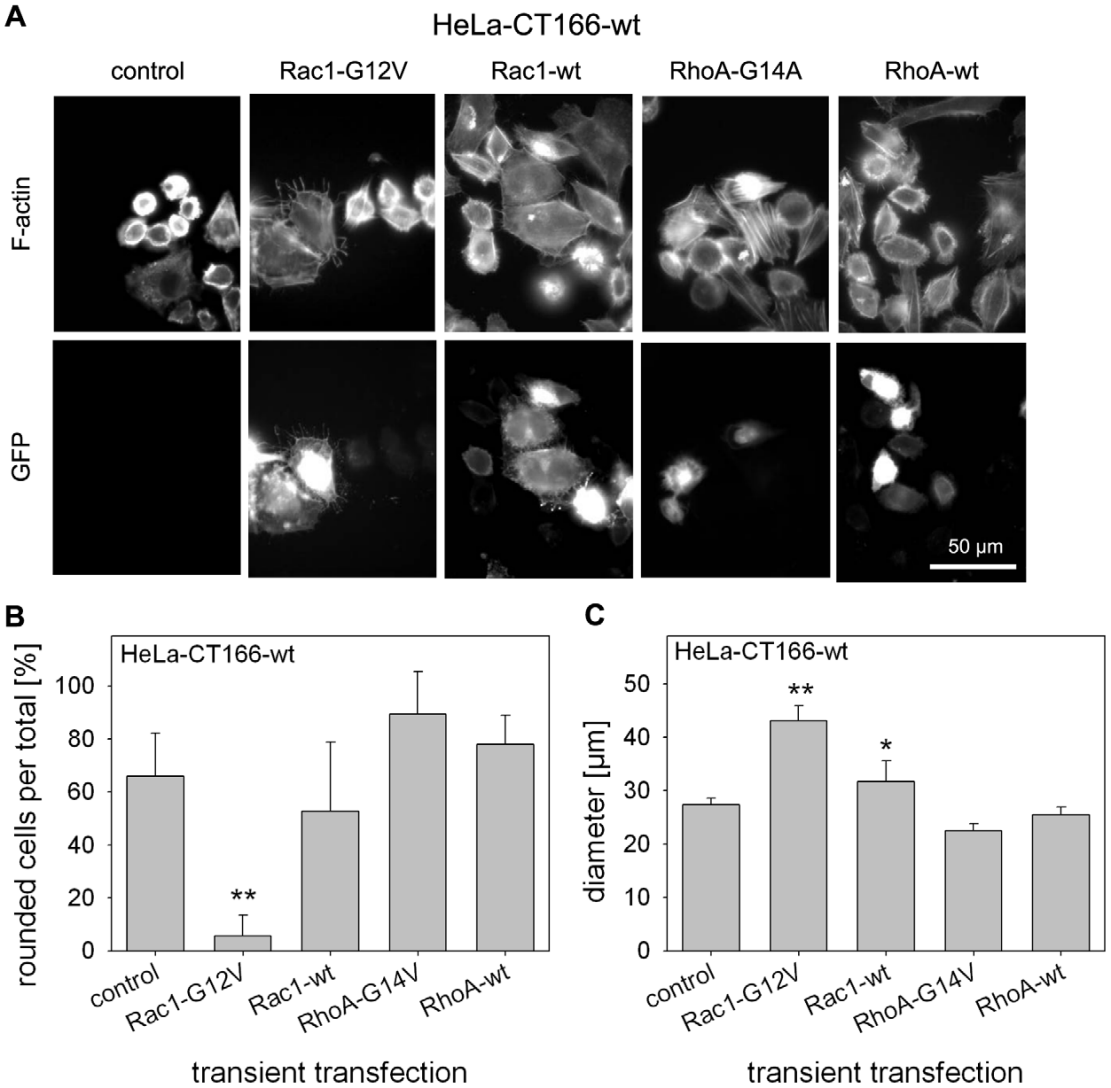
Expression of a constitutive active Rac1 mutant and over-expressed Rac1 reverts the CT166-induced phenotype. HeLa-CT166-wt, HeLa-CT166-mut and HeLa-control cells were transiently transfected with the pEGFP-C1 vector encoding for human Rac1-G12V or Rac1-wt, RhoA-G14V or RhoA-wt, respectively. The rhodamine-phalloidin stained actin cytoskeleton and expression of GFP-fusion protein were visualized by fluorescence microscopy (**A**). Rounded GFP-positive cells (cell diameter <30 µm) were quantified and given as the ratio of rounded per total GFP-positive cells in percent (** indicates significant less rounded cells as compared to control cells, p<0.005) (**B**). The average cell diameter of GFP-positive cells was determined (*/** indicates significant increase as compared to control cells, p<0.05/p<0.005) (**C**). Depicted is the mean ± SD of three independent experiments.

To show a critical role of Rho proteins in CT166-induced actin re-organization, constitutively active Rac1-G12V and Rac1-wildtype (Rac1-wt) were transiently transfected into HeLa-CT166-wt cells. The actin cytoskeleton was analyzed in HeLa-CT166-wt cells expressing EGFP-tagged Rac1-G12V or Rac1-wt (Figure 5). Expression of Rac1-wt moderately reduced CT166-induced cell rounding (Figure 5). By contrast, Rac1-G12V, that is refractory to glucosylation by TcdB [11], almost completely prevented CT166-induced actin re-organization (Figures 5A and B) and the decrease of the average cell diameter (Figure 5C). Expression of either Rac1-G12V or Rac1-wt did not interfere with CT166 expression in HeLa-CT166-wt cells, as the cellular CT166 level was comparable in the presence or absence of Rac1-G12V (Figure S5B). The difference in the protective effect of either Rac1-wt or Rac1-G12V supports the hypothesis that CT166 glucosylates Rho proteins, in particular Rac1: Ectopically expressed Rac1-wt can be glucosylated and is thus not capable of preserving Rac1 activity as efficaciously as the ectopically expressed non-glucosylable Rac1-G12V, an observation also reported for TcdB-catalyzed Rac1 glucosylation [13].

**Figure 6:**
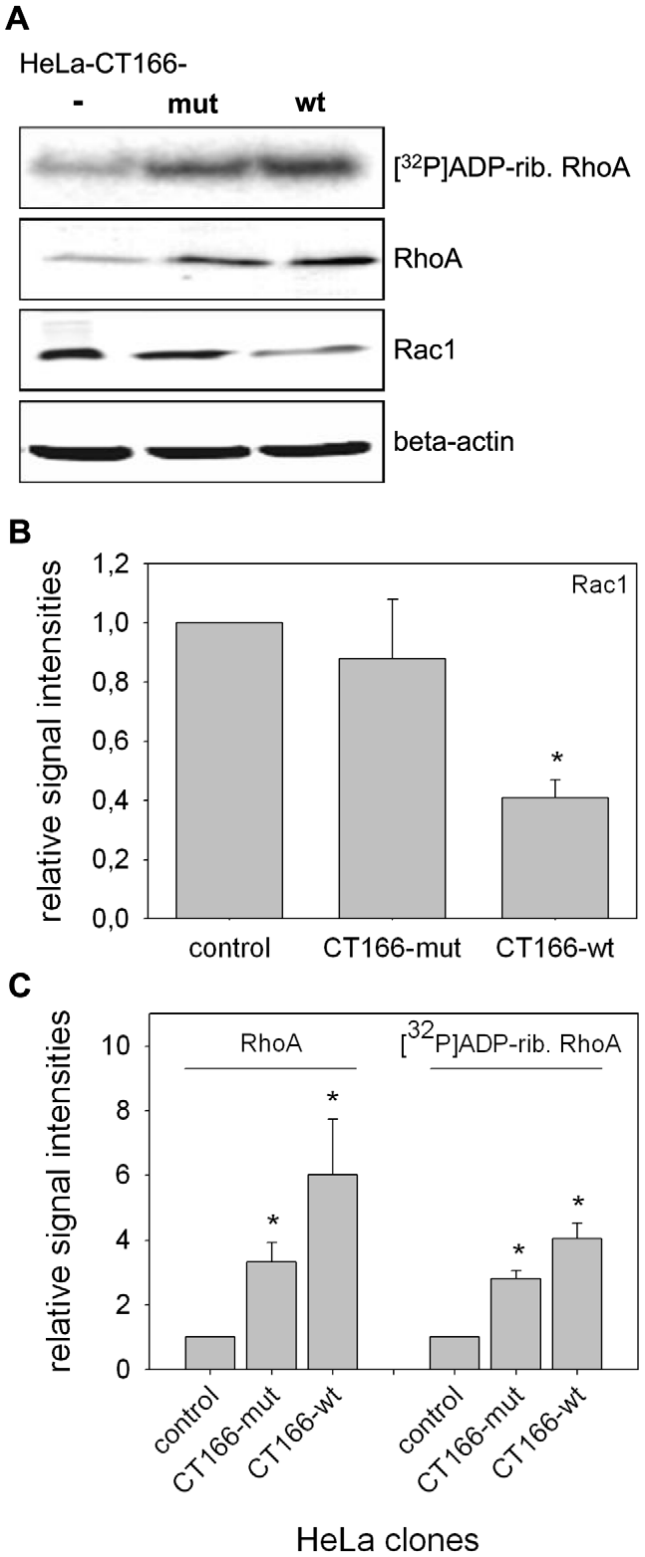
The Rac1 level is reduced, while RhoA is increased in CT166 expressing HeLa cells. Cellular protein levels of RhoA and Rac1 were analyzed in tetracycline-induced (1 µg/ml for 24 h) HeLa-CT166-wt, HeLa-CT166-mut or HeLa-control cells by Western blot analysis or sequential [^32^P]ADP-ribosylation (RhoA only). Representative Western blots and a representative autoradiography of [32P]ADP-ribosylated RhoA are presented (one out of three experiments with similar outcome is depicted) (**A**). Signal intensities from three independent experiments were quantified using Kodak software (B and C). The signal intensities of either RhoA or Rac1 from HeLa-control cells were set as 1.0. (* indicates statistically significant differences as compared with HeLa-control cells, p<0.05) (**B**).

Next, the cellular level of Rac1 was analyzed in HeLa-CT166-wt, HeLa-CT166-mut and in HeLa-control cells by Western blotting using anti-Rac1 (Mab23A8), which has been reported to be unaffected in its antigen recognition by the glucosylation state of Rac1. The Rac1 level was reduced in HeLa-CT166-wt cells compared to HeLa-CT166-mut and HeLa-control cells (Figure 6). This reduced Rac1 level impeded to directly check for CT166-induced Rac1 glucosylation at Thr-35 using the glucosylation-sensitive Rac1 antibody clone 102 [17]. The reduced Rac1 level may support the hypothesis that Rac1 is targeted by CT166. Degradation of Rho proteins has been suggested to represent a cellular response to limit cytotoxicity induced by toxin-catalyzed covalent modifications of Rho proteins [18]. In this line, RhoA degradation has been reported upon mono-ADP-ribosylation by C3-bot [19] or upon mono-glucosylation by TcdB [17]. It thus appears to be plausible that Rac1 (possibly covalently modified by CT166) was degraded in HeLa-CT166-wt cells. Treatment of these cells with the proteasome inhibitor MG132, however, resulted in cell death (unpublished observation) impeding further analysis.

CT166-induced actin re-organization and cell rounding was not prevented by ectopic expression of RhoA-G14V or RhoA-wildtype (Figure 5). This observation likely excludes a critical role of RhoA in CT166-induced actin re-organization. To check if CT166 targeted RhoA, the cellular level of RhoA was tracked by either Western blot analysis or sequential [^32^P]ADP-ribosylation of RhoA catalyzed by C3-bot [20]. The RhoA level was increased in HeLa-CT166-wt cells (less pronounced in HeLa-CT166-mut cells) compared to HeLa-control cells, as evidenced by either method (Figure 6). This observation likely excluded that RhoA from HeLa-CT166-wt cells was glucosylated at Thr-37, as RhoA glucosylation at Thr-37 would have caused decreased ADP-ribosylation of RhoA at Asn-41 [20]. Moreover, CT166-induced RhoA inactivation (e.g. by glucosylation) in HeLa-CT166-wt cells was also unlikely, for the reason that RhoA is critical for contractile ring formation in cytokinesis and thus critical for cell proliferation [21]. RhoA inactivation would have resulted in multinucleated cells (indicative of cytokinesis inhibition), which was not observed (Figure 3A).

**Figure 7:**
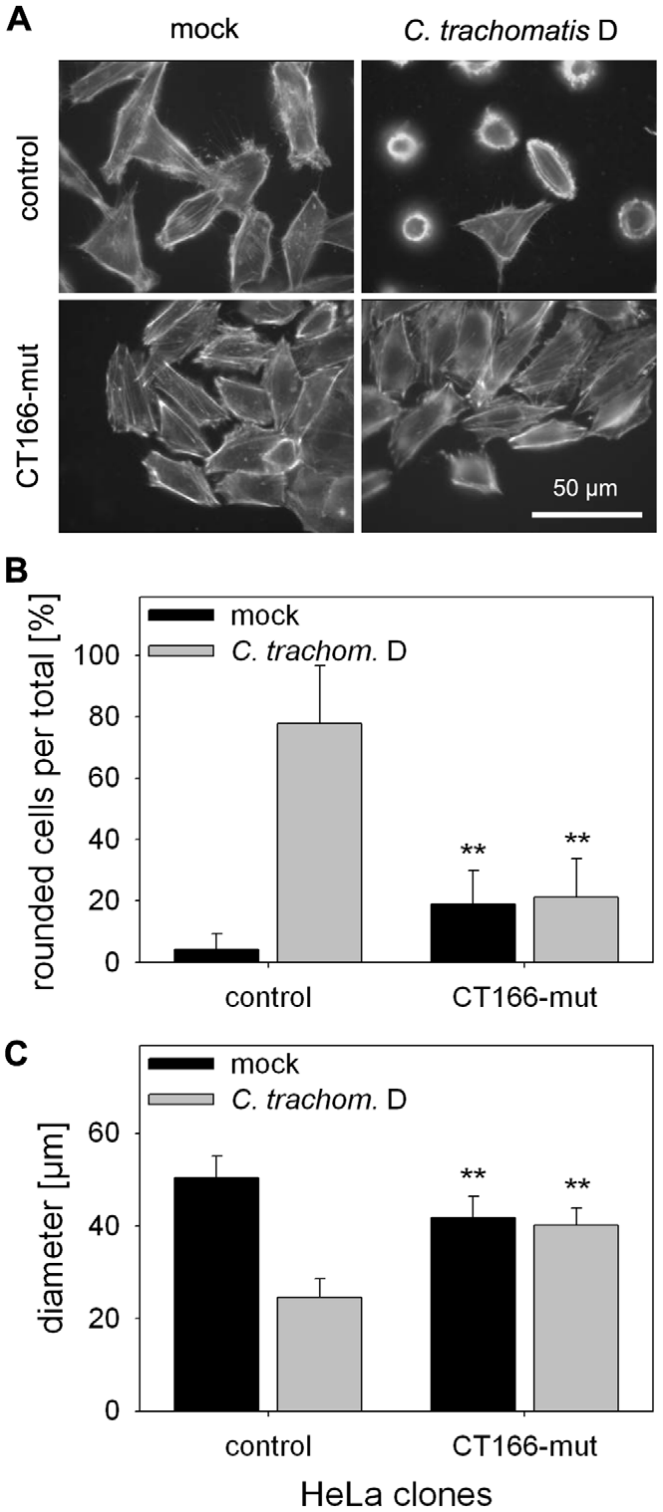
*C. trachomatis* serovar D induced cytopathic effects are less pronounced in CT166-mutant expressing cells. HeLa-CT166-mut cells and HeLa-control cells were infected with *C. trachomatis* D (MOI 100) or mock infected, following pre-treatment with 1 µg/ml tetracycline for 24 h. At 2 h post infection the cytopathic effects were less pronounced in CT166-mut expressing cells as determined by staining of the actin cytoskeleton with rhodamine-phalloidin (**A**) and by quantification of cell rounding. Rounded cells (cell diameter <30 µm) are given as ratio in percent (** indicates significant differences as compared to the corresponding control cells, p<0.005) (**B**). The average cell diameter was determined (** indicates significant differences as compared to the corresponding control cells, p<0.005) (**C**). Depicted is the mean ± SD of three independent experiments.

If CT166 targets Rac1, this targeting most likely includes first binding of the substrate Rac1 (and other possible substrate proteins from the Rho subfamily) to CT166. Binding of CT166-mut to Rho proteins may preserve substrate glucosylation by bacterial CT166 upon infection, and thus, may subsequently preserve actin re-organization induced by *C. trachomatis* D. HeLa-CT166-mut cells should thus exhibit a lower sensitivity to *C. trachomatis* D-induced actin re-organization compared to HeLa-control cells. This was in fact observed (Figure 7). These observations suggested that CT166-mut binds the CT166 substrate proteins from the Rho family and preserves them from CT166-induced glucosylation upon *C. trachomatis* D infection.

**Figure 8:**
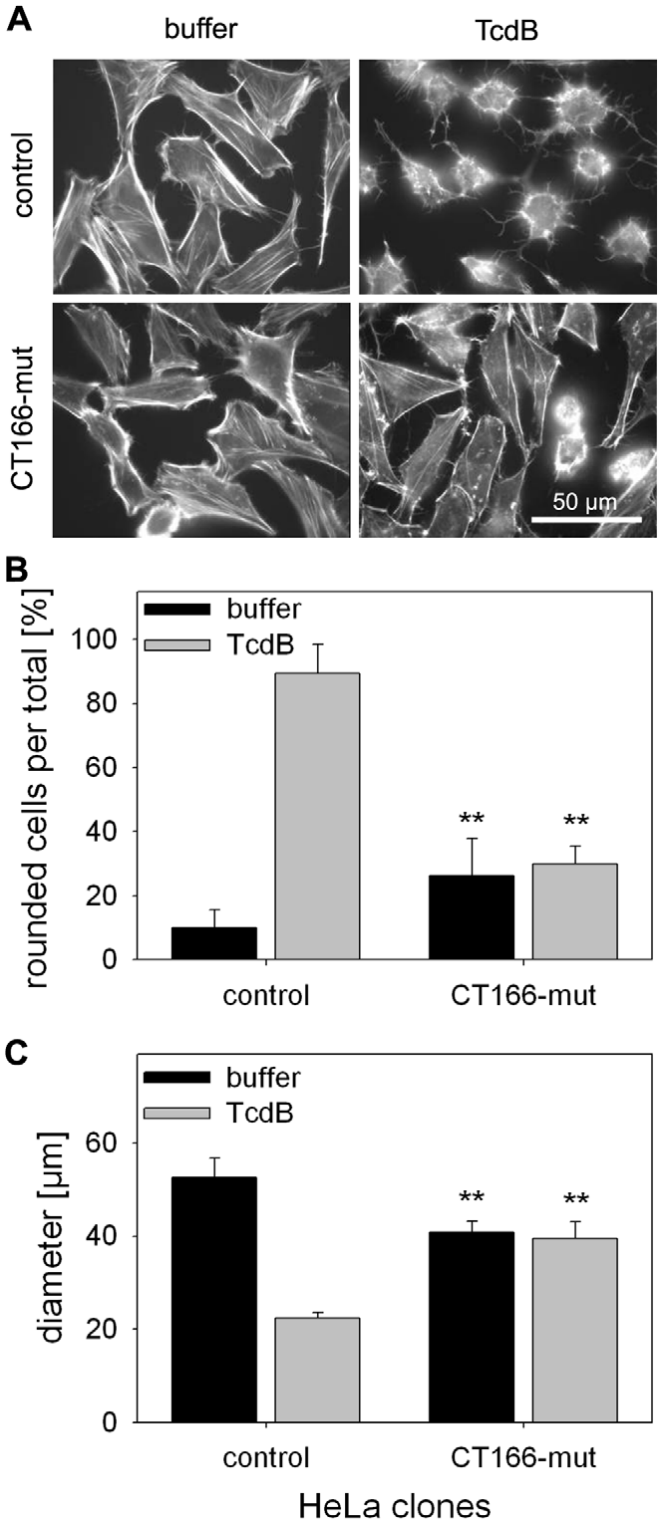
Expression of CT166-mutant delays cytopathic effects of TcdB in HeLa cells. HeLa-CT166-mut cells and HeLa-control cells were incubated with 1 µg/ml tetracycline for 24 h and subsequently with 0.3 ng/ml TcdB at 37°C for 2 h. The actin cytoskeleton was stained using rhodamine-phalloidin (**A**). Rounded cells (cell diameter <30 µm) were quantified and given as ratio in percent (** indicates significant differences as compared to the corresponding control cells, p<0.005) (**B**). The average cell diameter was determined (** indicates significant differences as compared to the corresponding control cells, p<0.005) (**C**). Depicted is the mean ± SD of three independent experiments. After 4 h incubation with TcdB, the HeLa-166-mut cells exhibited a phenotype comparable to TcdB-treated control cells (not shown).

Based on the assumption that CT166-mut binds cellular Rho proteins, HeLa-CT166-mut cells should also exhibit a lower sensitivity to TcdB-induced actin re-organization, through the preservation of Rho proteins from TcdB-induced glucosylation. And indeed, this was observed (Figure 8). The lower sensitivity of HeLa-CT166-mut cells was transient, as it was only observed after short-time treatment (2 h) with TcdB. After prolonged treatment (4 h) with TcdB the complete population of HeLa-CT166-mut cells was rounded (data not shown), likely due to the release of Rho proteins from the CT166-mut complex.

**Figure 9:**
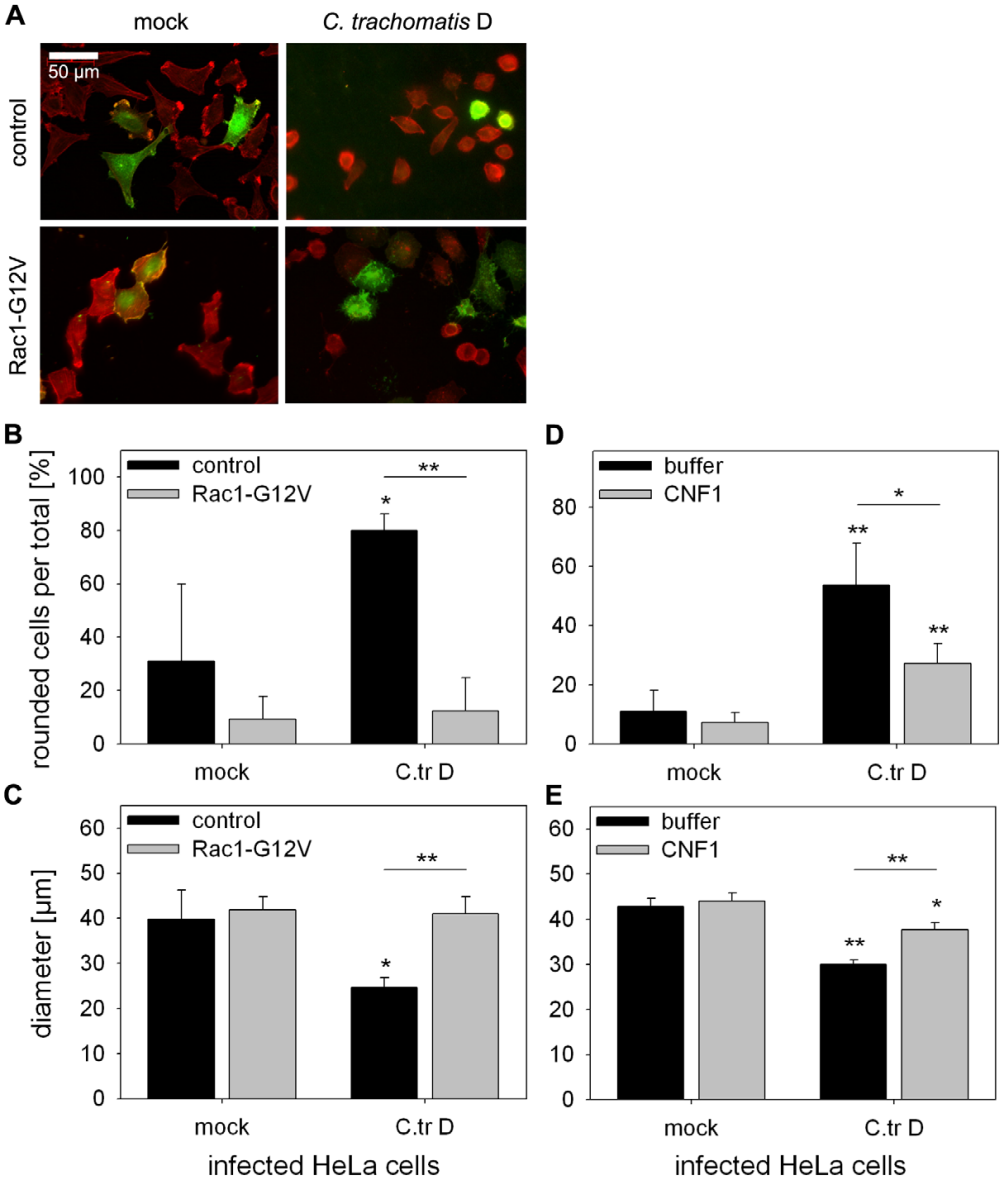
*C. trachomatis* D induced cytopathic effects are diminished by over-expression of a constitutive active Rac1 mutant, or by CNF1. HeLa cells were transiently transfected with the pEGFP-C1 vector encoding for human Rac1-G12V, or with the empty control vector. After 24 h the cells were either mock infected or infected with *C. trachomatis* D (MOI 100). Depicted is the rhodamine-phalloidin staining of the actin cytoskeleton and the fluorescence of GFP-fusion protein expressing cells at 2 h post infection (**A**). GFP-positive cells (cell diameter <30 µm) were analyzed for cell rounding (* indicates significant differences comparing mock-infected with *C. trachomatis* D-infected control cells, p<0.05; ** indicates significant differences comparing infected control cells with infected Rac1-G12V expressing cells, p<0.005) (**B**). The average cell diameter of GFP-positive cells was determined (* indicates significant differences comparing mock-infected with *C. trachomatis* D-infected control cells, p<0.05; ** indicates significant differences comparing infected control cells with infected Rac1-G12V expressing cells, p<0.005) (**C**). HeLa cells were treated with 0.1 µg/ml CNF1 from *E. coli* for 6 h, or with buffer. Subsequently, cells were either mock infected or infected with *C. trachomatis* D (MOI 100). At 2 h post infection the actin cytoskeleton was stained with rhodamine-phalloidin (not shown) and rounded cells (cell diameter <30 µm) were quantified and given as ratio in percent (** indicates significant differences as compared to the corresponding mock-infected cells, p<0.05; * indicates significant differences comparing infected control cells with infected CNF1-treated cells, p<0.05) (**D**). The average cell diameter was determined (*/** indicates significant differences as compared to the corresponding mock-infected cells, p<0.05/p<0.005; ** indicates significant differences comparing infected control cells with infected CNF1-treated cells, p<0.05) (**E**). Depicted is the mean ± SD of three independent experiments.

To finally provide evidence on an involvement of Rac1 in actin re-organization upon *C. trachomatis* D infection, actin re-organization induced by this serovar was analyzed in HeLa cells transfected with either GFP-tagged Rac1-G12V or the empty control vector. Consistent with above observation from HeLa-CT166-wt cells (Figure 5), expression of Rac1-G12V protected HeLa cells against *C. trachomatis* D-induced actin re-organization (Figure 9A, B, C). These findings confirm the critical role of Rac1 in *C. trachomatis* D-induced actin re-organization. HeLa cells that were pre-treated with CNF1 were also protected from *C. trachomatis* D-induced actin re-organization as compared to buffer-treated cells (Figure 9D, E). These observations suggest that the *C. trachomatis* D-induced actin re-organization is mediated by inactivation of the targeted Rho-proteins.

### CT166 as a Negative Regulator of Chlamydial Uptake into HeLa Cells

**Figure 10:**
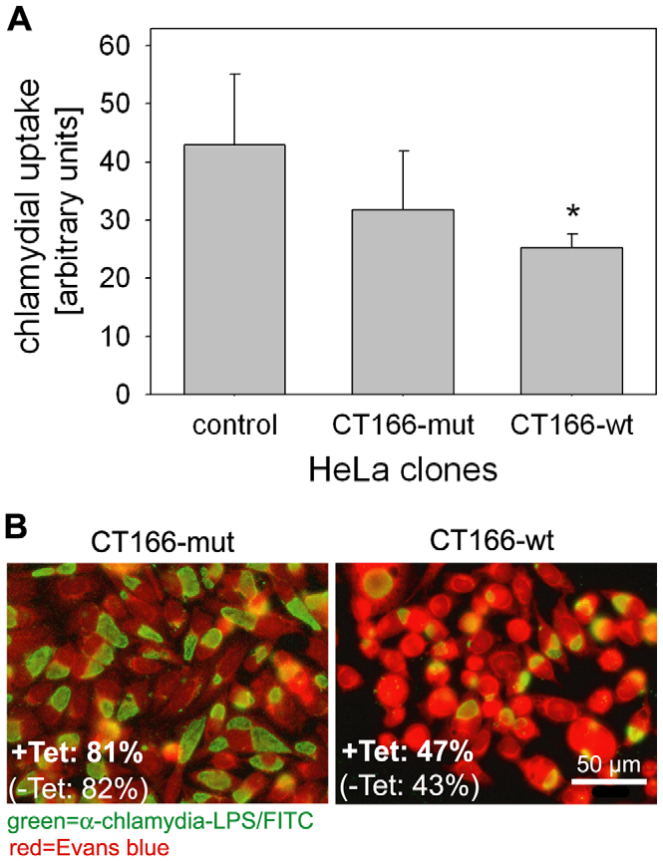
Uptake of *C. trachomatis* serovar D is impaired by CT166 expression. HeLa clones were infected with *C. trachomatis* D (MOI 3). Chlamydial uptake by HeLa-CT166-wt, HeLa-CT166-mut, or HeLa-control cells was determined by flow cytometry. Fluorescence intensity is expressed as arbitrary units as described in “Material and Methods”. Depicted is the mean ± SD of three independent experiments (* indicates significant differences as compared to control cells, p<0.05) (**A**). Immunofluorescence staining was performed at 24 h p.i. using anti-chlamydia-LPS IgG-FITC (green). Cells were stained with Evans blue (red) (**B**). Given is the ratio of infected cells per total cells in % in a representative experiment, comparing infected cells that were either pre-treated with 1 µg/ml tetracycline for 24 h (micrographs), or without.

Rac1 is a key host-cell protein regulating bacterial uptake. The bacterial effector CT166 may thus negatively regulate the efficiency of *C. trachomatis* D uptake into host-cells. *C. trachomatis* D infection was monitored by flow cytometry 2 h p.i. using a FITC labeled antibody directed against chlamydial LPS. Flow cytometry revealed a significant reduction of chlamydial uptake in HeLa-CT166-wt (but not in HeLa-CT166-mut) cells compared to HeLa-control cells (Figures 10A and S6). *C. trachomatis* D uptake into HeLa-CT166-wt or HeLa-CT166-mut cells was further analyzed using immunofluorescence microscopy after 24 h p.i. (Figure 10B). *C. trachomatis* D infected almost the complete population of HeLa-CT166-mut cells (>80% of total cells), similar as HeLa-control cells (data not shown). In contrast, the rate of infected HeLa-CT166-wt cells was clearly reduced (43–47%). The rates of *C. trachomatis* D infection were independent of tetracycline pre-treatment (Figure 10B), re-confirming that the low expression of CT166-wt due to the leakiness of the gene repression system was sufficient for the full CT166-wt activity. In course of *C. trachomatis* D infection, the effector protein CT166 likely participates in the control of *C. trachomatis* D uptake into host-cells through manipulation of Rac1 activity.

## Discussion

Our study is based on the observation that *CT166* ORF containing *C. trachomatis* strains such as serovar D cause actin re-organization comparable to that observed upon treatment with CGTs (Figure 1; [2]). The aim of our study was to clarify whether CT166 causes actin re-organization similar to CT166-expressing *C. trachomatis* strains and CGTs - and to elucidate the underlying biochemical mechanism. We therefore generated CT166-expressing HeLa cells. Due to the limitations to perform genetic manipulations on chlamydia, and technical problems using recombinant purified CT166 in a cell-free system, this is a successfully used alternative approach for the analysis of potential chlamydial effector proteins [22]. CT166 and CGTs share the DXD-motif, which is critical for the glucosyltransferase activity of many bacterial and mammalian glucosyltransferases [3], [23].

Thus, we additionally generated HeLa cell lines expressing a CT166-mutant (D415A.D417A) in order to investigate the role of the homologue DXD-motif for CT166 function. CT166-expressing cells exhibited actin re-organization comparable to the CGTs showing that CT166 alone actually has the potential to induce the cytoskeletal effects. The DXD-motif in CT166 is critical for this function, as the actin cytoskeleton was altered to a significantly minor extent in HeLa-CT166-mut cells. Comparable observations have been reported for the CGTs [3]. Similar results were obtained using independent clones, as well as transiently transfected cells, excluding clonal artifacts. Comparison of non-induced low-expressing HeLa-CT166-wt clones in the leaky repression system with strongly over-expressing tetracycline-induced clones revealed the same results. This high biological potential suggests an enzymatic activity of CT166. Moreover, this observation rules out that the different phenotypes of HeLa-CT166-wt and HeLa-CT166-mut are due to possible slight differences in the expression levels of the recombinant proteins (Western blot, Figure S1A).

Bacterial toxins (including bacterial effector proteins) modulate actin dynamics by either directly targeting actin (e.g. actin ADP-ribosylating toxins) or by targeting Rho family proteins (e.g. glucosylating toxins or bacterial GEF or GAP proteins). The prevention of CT166-induced actin re-organization by either CNF1 treatment or expression of Rac1-G12V suggests that CT166 targets Rho proteins rather than actin. This conclusion is based on the finding that actin re-organization induced by Rho-modulating toxins (TcdB, C3-bot), but not by actin targeting toxins (e.g. C2 toxin or latrunculin B) is responsive to CNF1 treatment or Rac1-G12V expression [13], [16], [19].

Over-expressed Rac1 and expression of constitutive active Rac1-G12V, but not RhoA and RhoA-G14V, reverted the phenotype of HeLa-CT166-wt cells, demonstrating that active Rac1 is sufficient to preserve cells from CT166-induced actin re-organization. CT166 expression levels were unaffected by over-expression of Rac proteins. We therefore conclude that CT166 inactivates Rac1, as the resulting cytopathic effect could be compensated by increasing the amount of intracellular wildtype Rac1. Intriguingly, this effect became stronger when the constitutively active and glucosylation-resistant mutant Rac1-G12V was over-expressed. This indicates that Rac1 may be a target of CT166 and strongly suggests that CT166 inactivates Rac1 by glucosylation.

Our data suggest that RhoA in HeLa-CT166-wt cells was non-glucosylated, as evidenced by sequential ADP-ribosylation. Thus, CT166 may target Rac1 but not RhoA. Among the CGTs, several isoforms that glucosylate Rac but not Rho are described including lethal toxin from *Clostridium sordellii* [6] or Toxin B from so called “variant” *C. difficile* strains [8], [24]. The determined substrate spectrum, i.e. glucosylation of Rac1 but not RhoA, is in accordance with the successful generation of a viable cell line stably expressing CT166 since RhoA inactivation would have resulted in inhibited contractile ring formation and interrupted cell division [21]. Rac1 is involved in the regulation of G1/S through induction of cyclin D1 expression during G1 phase [25], [26] and G2/M progression through activation of the mitotic kinase Aurora [27]. Given that CT166 glucosylates and inactivates Rac1, delayed cell cycle progression may offer an explanation for the decreased proliferation rate of CT166-wt-expressing cells.

HeLa-CT166-mut cells were less affected by *C. trachomatis* D-induced actin-reorganization compared to control cells, similar as HeLa-CT166-mut cells were less affected by TcdB treatment. These results of the infection experiment are in good accordance with our assumption that CT166 targets Rho proteins, in particular Rac1. Given that CT166-mut binds Rac1 and temporarily prevents its covalent modification, this observation indicates that *C. trachomatis* D-induced actin re-organization includes Rac1 during infection. HeLa cells expressing the constitutively active Rac1-G12V were protected from *C. trachomatis* D-induced actin re-organization, confirming the critical role of Rac1 in *C. trachomatis* D-induced actin re-organization. Moreover, HeLa cells that were pre-treated with CNF1 were also protected against *C. trachomatis* D-induced actin re-organization. These observations suggest that the *C. trachomatis* D-induced cytopathic effect is mediated by inactivation of the targeted Rho-proteins. Moreover, these data provide direct evidence that endogenous CT166 is translocated into the host-cell cytosol upon infection.

Chlamydial uptake was significantly impaired in CT166-wt-expressing cells. We used flow cytometry at 2 h p.i. in addition to the commonly used immunofluorescence staining (24 h p.i.), as flow cytometry turned out to be more sensitive for the detection of chlamydiae early in infection (data not shown). The results of both techniques are in good agreement: Both types of analysis clearly indicate that ectopic expression of CT166 diminishes chlamydial uptake.

Actin polymerization during chlamydial entry might be achieved involving Rac-dependent as well as -independent pathways, similar as multiple modes of induction of actin polymerization are observed with Salmonella. As an example, the chlamydial effector “translocated actin recruiting phosphoprotein” Tarp has been identified to promote actin polymerization by directly nucleating actin [22], [28] or by activating Rac-activating guanine nucleotide exchange factors [29]. Regarding chlamydial entry, the requirement of Rac1 activation and actin polymerization has been demonstrated for *C. trachomatis* L2 entry [30]–[32], and knock-down of Rac1 (but not RhoA) decreases infectivity determined at 24 h p.i. [33]. Rac1 (and Cdc42) was also activated upon entry of *C. caviae*, a species that possesses an ORF with homology to CGTs (consensus sequence: [4]; alignment: [2]), but neither Rac1 (nor Cdc42) was required to initiate actin polymerization [34]. Moreover, a subsequent decrease of Rac1 has been detected in *C. caviae*-infected cells [34]. In the meantime, it turned out that the antibody which was used (clone 102) binds only the non-glucosylated form of Rac1 [17] suggesting *C. caviae*-induced Rac1 glucosylation. In this case it might be interesting to determine the ratio of non-glucosylated Rac1 to total Rac1 in future studies.

It has been shown that serovar D also activates Rac1 [30], but the necessity of Rac1 activation has not been proven for the entry of *C. trachomatis* serovars containing ORFs with homology to CGTs. Our findings support the involvement of Rac1 in the uptake of *C. trachomatis* D.

Our approach delivers important data about the biochemical characterization of CT166 and its biological potential. However, it should be noted that this characterization of CT166 is based on over-expression of the protein. Moreover, chlamydial infections with high MOI only, cause drastic, easily observable, general actin re-organization and cell rounding. On the other hand, CT166 seems to be biologically highly potent, e.g. concluded from the observation that the repressed but leaky expression system caused already almost complete cell responses. Taking these points into consideration it might still be justified to speculate on the pathophysiological role of CT166 in chlamydial infection at low MOI: The involvement of Rac1-activation in chlamydial entry suggests the requirement of its subsequent inactivation to limit excessive actin polymerization. Upon bacterial uptake, factors such as the effector Salmonella protein tyrosine phosphatase (SptP) antagonize actin polymerization by inactivating Rac (and Cdc42) in order to rebuild host-cell morphology afterwards [35]. But so far, no chlamydial effector protein has been identified that targets cellular components which antagonize actin polymerization in order to recover normal host-cell morphology. It is tempting to speculate that CT166 might have a similar, locally limited, Rac1-antagonizing function as SptP. The presence of CT166 in EBs and in the host-cell cytosol during the first 60 min p.i. [2] together with the diminished uptake of chlamydiae in CT166 over-expressing HeLa cells further support the assumption that CT166 plays a role during the controlled entry of this pathogen.

In summary, the biological activity of CT166 was analyzed in HeLa cells stably expressing CT166. The prominent observation was that the actin cytoskeleton was re-organized in HeLa-CT166-wt cells. In particular, the loss of stress fibres, lamellipodia and filopodia and finally the loss of cell shape (cell rounding) were observed. These morphological changes were comparable to those observed upon treatment with TcdB, a toxin that mono-glucosylates Rho family proteins [8]. CT166-induced actin re-organization resembled TcdB-induced actin re-organization in further aspects: (i) actin re-organization was prevented by either CNF1, a broad spectrum activator of Rho proteins, or ectopic expression of Rac1-G12V, and (ii) actin re-organization depended on the DXD-motif present on CT166 or TcdB. The latter observation suggests that CT166 is a glucosyltransferase, because the DXD-motif is characteristic for mammalian and bacterial glycosyltransferases [3], [23]. Our experiments provide strong evidence that CT166 acts as an effector protein inactivating Rac1 but not RhoA, most likely by glucosylation, ensuring a balanced uptake of *C. trachomatis* D during host-cell entry.

## Materials and Methods

### Cloning and Mutation of *CT166*

The open reading frame of *CT166* was amplified by polymerase chain reaction (PCR) using genomic DNA of *C. trachomatis* D/UW-3/Cx as the template. The PCR was carried out with *Pfu* DNA polymerase (Stratagene, Amsterdam, Netherlands) following the manufacturer's instructions. *CT166* was ligated into the prokaryotic expression plasmid pGEX-2T (GE Healthcare, UK) and the eukaryotic expression plasmids pcDNA4™/TO/*myc*-His B (Invitrogen, Karlsruhe, Germany) and pCS2+MT (kindly provided by A. Gossler, Hannover, Germany). For the creation of the pcDNA4™/TO/*myc*-His B construct, the stop codon of *CT166* was deleted in order to allow C-terminal fusion with the provided tags. The pCS2+MT construct was created in order to obtain N-terminal fusion with the 6x-myc-tag. Mutagenesis of the DXD-motif (D415A.D417A  =  CT166-mut) of all constructs was carried out using the QuickChange Site-Directed Mutagenesis Kit (Stratagene). The integrity of all constructs was confirmed by sequencing.

### Expression of Recombinant Proteins

*Escherichia coli* BL21 were transformed with pGEX-2T_CT166-wt or pGEX-2T_CT166-mut. Expression was induced with IPTG (Sigma, Deisenhofen, Germany). The expressed GST fusion proteins were isolated by affinity chromatography with glutathione-Sepharose (Pharmacia, Germany). Then, the proteins were recovered from the GST-tag by thrombin cleavage and thrombin was removed by binding to benzamidine-Sepharose. Expression was analyzed by SDS-PAGE and confirmed with mass spectrometry.

### Antiserum

One hundred micrograms of purified recombinant CT166-mut were dissolved in 250 µl PBS and mixed with 250 µl Freund's complete adjuvant (Sigma). The mixture was homogenized by sonication and administered to a rabbit by subcutaneous injection. The antibody titer was monitored by ELISA using the recombinant protein as antigen. The antiserum was pre-adsorbed to lysates of HeLa cells that were neither infected nor transfected, and then it was used for the detection of CT166 in Western blot analysis.

### Cell Cultures

Human cervical epithelial HeLa cells (ATCC; subclone kindly provided by R. Heilbronn, Berlin, Germany) were cultured in Earle's minimal essential medium (MEM) supplemented with 10% fetal calf serum, 2 mM glutamine, 0.1 M nonessential amino acids, and 1 mM sodium pyruvate (PAA Laboratories, Pasching, Germany). Native cells were grown at 37°C and 5% CO_2_. T-Rex™-HeLa cells (Invitrogen, Karlsruhe, Germany) were cultured according to the manufacturer's instructions in T-Rex medium (MEM with 10% fetal calf serum and 5 µg/ml Blasticidin (Invitrogen).

### HeLa T-Rex Clones

HeLa T-Rex™ cells were transfected with pcDNA4™/TO/*myc*-His_CT166-wt or pcDNA4™/TO/*myc*-His_CT166-mut, using Magnet Assisted Transfection (Iba, Goettingen, Germany). Stable clones were selected in the presence of 400 µg/ml Zeocin (Invitrogen). Limiting dilution was performed in order to obtain homogenous populations. Proper expression and inducibility with tetracycline were verified by Western blot analysis. Homogenous expression in the selected clones was confirmed by immunofluorescence staining using mouse anti-myc (Clontech, France) and anti-mouse IgG-FITC (DakoCytomation, Glostrup, Denmark). To obtain cells for control experiments HeLa T-Rex cells were transfected with pcDNA4™/TO/*myc*-His without insert. For the analyses, one clone of each of three cell lines was combined to obtain cell “sets”; i.e. set 1: HeLa-CT166-wt clone JT30, HeLa-CT166-mut clone JT4N, HeLa-control clone JT3; set 2: HeLa-CT166-wt clone JT26, HeLa-CT166-mut clone JTB4, HeLa-control clone JT1.

### Determination of Cell Proliferation

Growth of HeLa clones was monitored by fluorescence staining of the DNA with Hoechst 33258 (Sigma). Equal numbers of cells were seeded on a 96-well plate for each time point. The staining was performed with 20 µg/ml Hoechst 33258 for 30 min at 37°C and 5% CO_2_. Then fluorescence was measured (355 nm/460 nm, 1.0 s) using a Victor^3^ Multilabel Counter (PerkinElmer, Inc., Fremont, CA, USA). The corresponding number of cells was determined in comparison to a dilution series with known cell numbers.

### Chlamydial Culture

*Chlamydia trachomatis* serovar D/UW-3/Cx (ATCC; VR-885) was propagated in HeLa cells. For stock production, HeLa cell monolayers were infected with *C. trachomatis* serovar D EBs by centrifugation (55 min; 35°C; 2000×*g*) in Panserin 401 medium (Cytogen, Berlin, Germany) and 1 µg/ml cycloheximide (Sigma). Infected cells were cultivated at 37°C and 5% CO_2_ and EBs were harvested 2 days p.i.. The infected cells were destroyed mechanically. Cell debris was removed by a centrifugation step (15 min; 500×*g*). Then, the EBs in the supernatant were collected by centrifugation at 22,000×*g* for 1 h. EBs were washed with transport medium (1x PBS including 6.86% saccharose, 40 µg/ml Gentamicin, 0.002% Phenol red, 2% FCS), and collected again by centrifugation.

### SDS-PAGE

Sodium dodecyl sulfate polyacrylamid gel electrophoresis was performed with 10% polyacrylamid gels according to Maniatis' manuals [36]. Equal amounts of protein were loaded to each lane. The protein concentration of the lysates was determined using the ProtaQuant-Assay Kit (Serva, Heidelberg, Germany). For a protein standard, Precision Plus Protein Dual Color Standard (BioRad Laboratories, USA) was used.

### Western Blotting

Proteins were separated by SDS-PAGE and transferred onto a nitrocellulose membrane for 45 min at 13 V, using the Trans-Blot SD Semi-Dry Transfer Cell (BioRad). The membrane was blocked for 1 h with 5% (w/v) nonfat dried milk in TBS containing 0.1% Tween (0.1% TBST). Primary antibodies were applied over night at 4°C as follows: anti-CT166 antiserum, diluted 1∶500 in blocking solution; mouse anti-Rac1 (Mab23A8; Millipore, Billerica, USA), diluted 1∶1000 in 0.05% TBST; mouse anti-RhoA (26C4; Santa Cruz Biotechnology, Santa Cruz, CA, USA), diluted 1∶300 in 0.05% TBST; and mouse anti-beta-actin (AC-40, Sigma), diluted 1∶2000 in 0.05% TBST. The membrane was washed and incubated for 2 h at room temperature either with anti-rabbit IgG-HRP (GE Healthcare), diluted 1∶1000 in 0.1% TBST containing 5% BSA, or anti-mouse IgG-HRP (MP Biomedicals, Illkirch, France), diluted 1∶2000 in 0.5% TBST containing 5% nonfat dried milk. Then, the membrane was washed again and developed with SuperSignal West Pico Chemiluminescent Substrate (Pierce, Rockford, IL, USA). Chemiluminescence was detected using the Intelligent Dark Box LAS-3000 (Fujifilm Life Science, Stamford, CT, USA).

### Cytoskeletal Staining

Cells were grown on glass coverslides and processed (i.e. infected, transiently transfected, or stimulated) as indicated. The cells were washed with PBS and fixed with 4% paraformaldehyde for 15 min. Cells were treated with 0.3% Triton X-100 in PBS for 5 min and blocked with 5% BSA for 60 min at room temperature. Staining of the actin cytoskeleton was performed with rhodamine-conjugated phalloidin (Molecular Probes, Eugene, OR, USA) for 45 min at room temperature. Subsequently, nuclei were occasionally stained with DAPI in PBS containing 0.1% Tween-20 for 15 min. Coverslides were washed and mounted with Antifade (Invitrogen). The cells were analyzed using a BX60 fluorescence microscope (Olympus, Hamburg, Germany) or a Zeiss Axiovert 200 M (Zeiss, Goettingen, Germany).

### Quantification of Actin Re-organization

Cell rounding was determined by phase contrast microscopy (Axiovert 35; Zeiss) and given as the ratio of rounded per total cells. The cell diameter of each cell was determined by measuring the longest distance of the rhodamine-phalloidin-stained cells using KappaImageBase software. Cells exhibiting a cell diameter <30 µm were classified as “rounded”.

### Transient Transfection Experiments

For the Rho GTPase complementation experiments HeLa clones were transfected with plasmids encoding for human RhoA-wt, RhoA-G14V, Rac1-wt, Rac1-G12V cloned into the pEGFP-C1 vector using FuGENE® (Roche, Basel, Switzerland), according to the provider's manual. Staining of the actin cytoskeleton occurred as described above (see “Cytoskeletal Staining”), or cells were lysed and subjected to SDS-PAGE and Western blot analysis. Infection of Rac1-G12V-transfected HeLa cells was performed 24 h after transfection as described below (see “Infection Experiments”). For analysis of HeLa cells transiently expressing CT166-wt or CT166-mut, cells were transfected with pCS2+MT_CT166 or pCS2+MT_CT166-D415A.D417A using Magnet Assisted Transfection (Iba). After incubation over night at 37°C and 5% CO_2_, the cells were fixed with 4% paraformaldehyde for 5 min. The expression of the recombinant proteins was determined by immunofluorescence using mouse anti-myc (Clontech, France) and anti-mouse IgG-FITC (DakoCytomation, Glostrup, Denmark) and staining of the actin cytoskeleton occurred as described above (see “Cytoskeletal Staining”).

### Infection Experiments

HeLa cells were grown on glass coverslides, and further processed prior to infection as indicated in the corresponding text. For experiments using HeLa clones either protein expression was induced by incubation with 1 µg/ml tetracycline for 24 h, or cells were left untreated as indicated in the text. Infection with *C. trachomatis* D occurred by centrifugation for 55 min at 35°C and 2,000×*g* in HeLa T-Rex medium (MOI as indicated in the text). Since chlamydiae are susceptible to tetracycline, the cells were rigorously washed before infection. Control infections of tetracycline treated HeLa cells showed that this procedure was sufficient to permit normal infection rates and chlamydial development (data not shown). After centrifugation, the cells were washed again and cultured at 37°C and 5% CO_2_. For determination of host-cell morphology actin staining was performed at indicated time points as described above (see “Cytoskeletal Staining”). For chlamydiae detection the infected cells were washed and fixed with methanol at −20°C for 10 min. at 24 h p.i.. Immunofluorescence staining was carried out with anti-chlamydia LPS IgG-FITC (DakoCytomation) at 35°C for 30 min and cells were stained with Evans blue (Sigma).

### Flow Cytometry

HeLa clones were infected with MOI 3 as described above (see “Infection Experiments”). The infected cells were trypsinized at 2 h p.i., pelleted by centrifugation, and fixed with Cellfix (BD Biosciences). Each sample was divided in order to allow extracellular staining and staining of total chlamydiae. Staining of total (intracellular plus remaining extracellular) chlamydiae occurred with anti-chlamydia LPS IgG-FITC (BioRad, Hercules, CA, USA) after blocking and permeabilization with 3% BSA in PBS and 0.1% saponin for 45 min on ice. PBS with 0.5% fetal calf serum and 0.1% saponin was used for washing. Cells were fixed with Cellfix and flow cytometry was performed using a FACSCalibur cytometer (BD Biosciences). Control staining of potentially extracellular chlamydiae was performed in the absence of saponin. To determine chlamydial uptake by flow cytometry (10.000 cells per sample) the acquired fluorescent signals from the non-infected control was substracted from the sample of interest to exclude nonspecific signals. The signal intensities from the extracellular sample were substracted from the corresponding total stained sample to determine the fluorescence signal that derived from intracellular chlamydiae. This signal was proportionally correlated to the whole fluorescence intensity of the performed measurement and expressed as arbitrary units (a), whereby the whole fluorescence intensity was set to 100.

### TcdB Treatment of HeLa Cells

HeLa-CT166-mut and control cells were grown in 12-well plates to sub-confluency and pre-treated with 1 µg/ml tetracycline for 24 h. Then cells were incubated with 0.3 ng/ml TcdB [10] at 37°C. HeLa cells were treated with 0.3 ng/ml or 1 ng/ml TcdB for 2 h. Staining of the actin cytoskeleton occurred as described above (see “Cytoskeletal Staining”), or cells were lysed and subjected to SDS-PAGE and Western blot analysis.

### CNF1 Treatment of HeLa Cells

HeLa-CT166-wt cells and control cells were grown to sub-confluency and pre-treated with 1 µg/ml tetracycline for 24 h. Cytotoxic necrotizing factor 1 (CNF1, kindly provided by Gudula Schmidt, Freiburg) from *E. coli* was purified as previously described [14]. Cells were incubated with 0.1 µg/ml CNF1 at 37°C for 6 h. Staining of the actin cytoskeleton occurred as described above, or cells were lysed and subjected to SDS-PAGE and Western blot analysis. HeLa cells were incubated with 0.1 µg/ml CNF1 at 37°C for 6 h and proceeded to infection and subsequent staining of the actin cytoskeleton (see above: “Infection Experiments”; “Cytoskeletal Staining”).

### Sequential [^32^P]ADP-ribosylation Reaction

[^32^P]ADP-ribosylation assay was performed as described [37]. In brief, soluble fractions of HeLa clones were incubated with *C. botulinum* C3 exoenzyme in the presence of 0.3 µM [^32^P]NAD (PerkinElmer, Inc., Bosten, USA) for 30 min at 37°C. *C. botulinum* C3 exoenzyme was expressed as GST-fusion protein in *E. coli*. The reaction was terminated by addition of Laemmli sample buffer. Subsequently, the samples were separated by SDS-PAGE and subjected to PhosphoImager (Cyclone, Packard, Groningen, Netherlands) analysis.

### Statistical Analysis

Statistical evaluation of data was performed using a two-sided Student *t*-test. P-values<0.05 (*) were considered statistically significant. P-values<0.005 were indicated by two asterisks (**).

### Ethics Statement and Biosafety-Consideration

According to the German gene technology law the designated project is documented with the reference number Klos/11/01.02.2005 and Klos/12/01.06.2005. The work was done under biosafety level 1 conditions. A statement of the local ethics committee is not required.

## Supporting Information

Figure S1 HeLa clones express similar amounts of CT166-wt or CT166-mut as demonstrated by Western blot, and two sets of HeLa clones exhibit a similar phenotype. Two sets of clones of the generated cell lines HeLa-CT166-wt and HeLa-CT166-mut and non-expressing control cells were incubated with 1 µg/ml tetracycline for 24 h (+) or left untreated (−). The same amount of protein of each lysate was subjected to SDS-PAGE and CT166 expression was analyzed by Western blot, using anti-CT166 antiserum. Additionally, beta-actin was determined for a loading control in set 2 (A). Application of 5 instead of 1 µg/ml tetracycline did not further increase CT166 expression (B). One representative Western blot analyzing one of two independent clones for each generated cell line is depicted. The actin cytoskeleton of two independent sets of the generated cell lines HeLa-CT166-wt and HeLa-CT166-mut and of control cells was stained with rhodamine-phalloidin, after 24 h incubation with 1 µg/ml tetracycline. Both CT166-wt expressing clones exhibit actin re-organization and a rounded phenotype (HeLa clones without tetracycline exhibit a similar phenotype; data not shown) (C). Rounded cells (cell diameter <30 µm) of set 2 HeLa clones were quantified by microscopy and are presented as the ratio of rounded cells in percent. Comparison of untreated and tetracycline-treated HeLa clones reveals similar results (D). The average cell diameter was determined in set 2 HeLa clones (E). Depicted is the mean ± SD of three independent experiments. (** indicates significant difference compared to control cells, p<0.005.)
(1.44 MB TIF)

Figure S2 HeLa clones homogenously express CT166-wt or CT166-mut. HeLa-CT166-wt, HeLa-CT166-mut and non-expressing control cells were incubated with 1 µg/ml tetracycline for 24 h. The actin cytoskeleton was stained with rhodamine-phalloidin. CT166-wt and CT166-mut expression was determined by immunofluorescence detecting the C-terminal myc-tag using mouse anti-myc and anti-mouse IgG-FITC.
(1.72 MB TIF)

Figure S3 Cytopathic effect in HeLa cells transiently expressing CT166. HeLa cells were transiently transfected to express myc-tagged CT166-wt or CT166-mut. Expression of the recombinant proteins was determined by immunofluorescence using mouse anti-myc and anti-mouse IgG-FITC. Morphological changes of the actin cytoskeleton stained with rhodamine-phalloidin are already visible in low CT166 expressing HeLa cells. One representative experiment of three is depicted.
(2.14 MB TIF)

Figure S4 CNF1 prevents TcdB-induced morphological changes in HeLa cells. HeLa cells were incubated with 15 µg/ml CNF1 from *E. coli* for 6 h, or with 1 ng/ml TcdB for 2 h, or with CNF1 for 6 h followed by TcdB for another 2 h, as indicated.
(1.28 MB TIF)

Figure S5 Neither CNF1 or TcdB treatment, nor Rac1 over-expression reduces CT166-wt expression. HeLa-CT166-wt cells were incubated with 1 µg/ml tetracycline for 24 h. Subsequently, cells were incubated with 15 µg/ml CNF1 from *E. coli* for 6 h, or with 1 ng/ml TcdB for 2 h, or with CNF1 for 6 h followed by TcdB for another 2 h, as indicated (A). HeLa-CT166-wt cells were transiently transfected with the pEGFP-C1 vector encoding for human Rac1-G12V or Rac1-wt, respectively, or the empty control vector following incubation with 1 µg/ml tetracycline for 24 h. Another 24 h after transfection, lysates were prepared (B). Lysates were analyzed for CT166 expression by Western blot, using anti-CT166 antiserum. Beta-actin was determined for a loading control.
(0.26 MB TIF)

Figure S6 Uptake of *C. trachomatis* serovar D is impaired by CT166 expression - flow cytometry histogram supporting Figure 10A. HeLa clones were infected with *C. trachomatis* serovar D (MOI 3). Chlamydial uptake by HeLa-CT166-wt, HeLa-CT166-mut, or HeLa-control cells was determined by flow cytometry at 2 h p.i.. The x-axis of the depicted histograms presents the intensity of the anti-chlamydia-FITC signal; the y-axis presents the number of counts; grey histogram: without saponin (extracellular staining); bold line: with saponin (extra- and intracellular staining). The right panel (expressed as arbitrary units) is showing the fluorescence exclusively from intracellular chlamydiae. The calculation method allows to exclude non-specific signals from the host-cell and from extracellular chlamydiae bound to the cells surface. Depicted is one experiment as an example for the calculation method.
(0.11 MB TIF)
