# Supplementary figures and images for: Actin Re-Organization Induced by Chlamydia trachomatis Serovar D - Evidence for a Critical Role of the Effector Protein CT166 Targeting Rac
Source: PLoS One. 2010 Mar 25;5(3):e9887. doi: 10.1371/journal.pone.0009887 (PMC2845625; doi:10.1371/journal.pone.0009887)

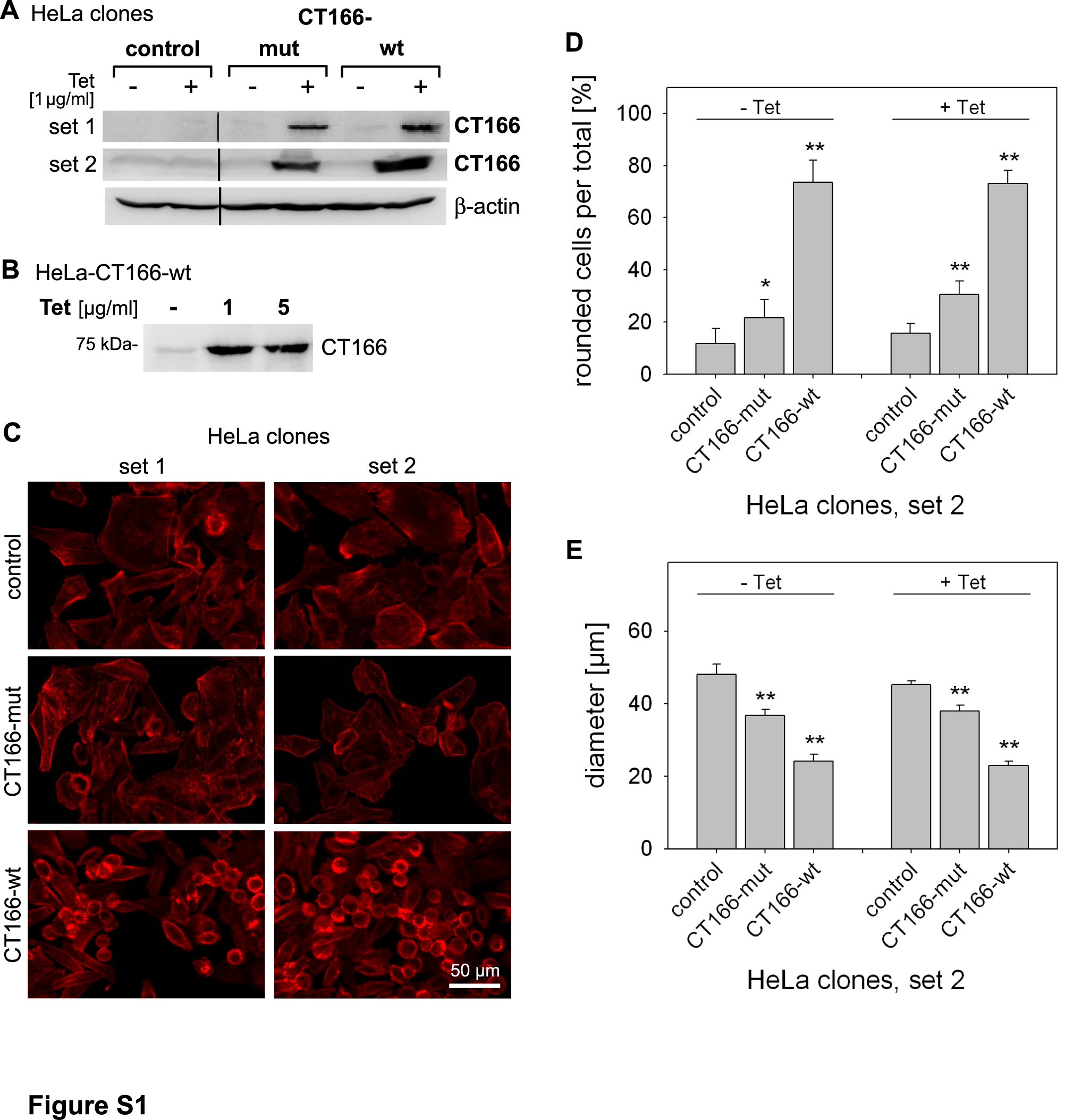

Supplement: Figure S1 — HeLa clones express similar amounts of CT166-wt or CT166-mut as demonstrated by Western blot, and two sets of HeLa clones exhibit a similar phenotype. Two sets of clones of the generated cell lines HeLa-CT166-wt and HeLa-CT166-mut and non-expressing control cells were incubated with 1 µg/ml tetracycline for 24 h (+) or left untreated (−). The same amount of protein of each lysate was subjected to SDS-PAGE and CT166 expression was analyzed by Western blot, using anti-CT166 antiserum. Additionally, beta-actin was determined for a loading control in set 2 (A). Application of 5 instead of 1 µg/ml tetracycline did not further increase CT166 expression (B). One representative Western blot analyzing one of two independent clones for each generated cell line is depicted. The actin cytoskeleton of two independent sets of the generated cell lines HeLa-CT166-wt and HeLa-CT166-mut and of control cells was stained with rhodamine-phalloidin, after 24 h incubation with 1 µg/ml tetracycline. Both CT166-wt expressing clones exhibit actin re-organization and a rounded phenotype (HeLa clones without tetracycline exhibit a similar phenotype; data not shown) (C). Rounded cells (cell diameter <30 µm) of set 2 HeLa clones were quantified by microscopy and are presented as the ratio of rounded cells in percent. Comparison of untreated and tetracycline-treated HeLa clones reveals similar results (D). The average cell diameter was determined in set 2 HeLa clones (E). Depicted is the mean ± SD of three independent experiments. (** indicates significant difference compared to control cells, p<0.005.) (1.44 MB TIF) [file pone.0009887.s001.tif]

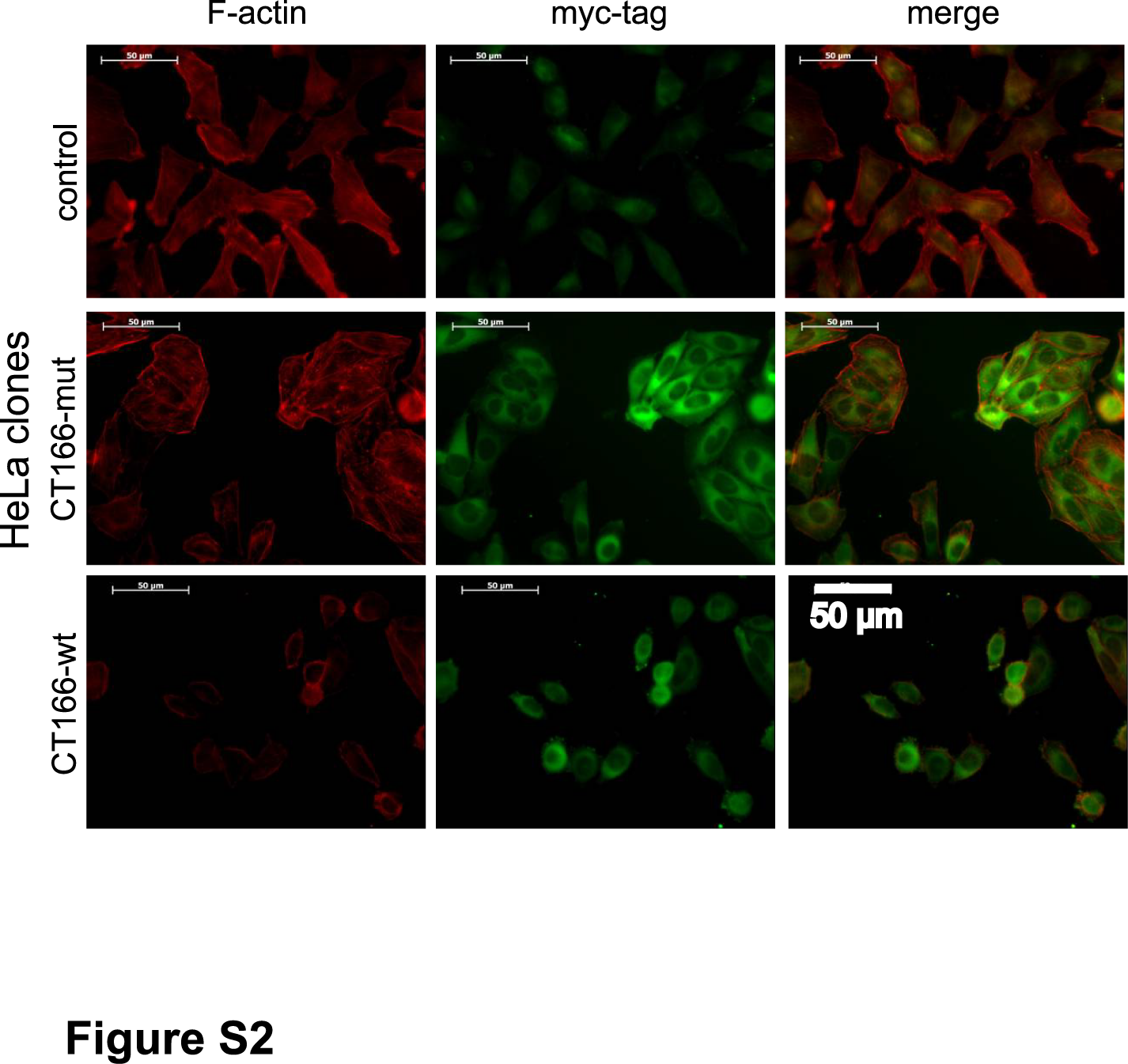

Supplement: Figure S2 — HeLa clones homogenously express CT166-wt or CT166-mut. HeLa-CT166-wt, HeLa-CT166-mut and non-expressing control cells were incubated with 1 µg/ml tetracycline for 24 h. The actin cytoskeleton was stained with rhodamine-phalloidin. CT166-wt and CT166-mut expression was determined by immunofluorescence detecting the C-terminal myc-tag using mouse anti-myc and anti-mouse IgG-FITC. (1.72 MB TIF) [file pone.0009887.s002.tif]

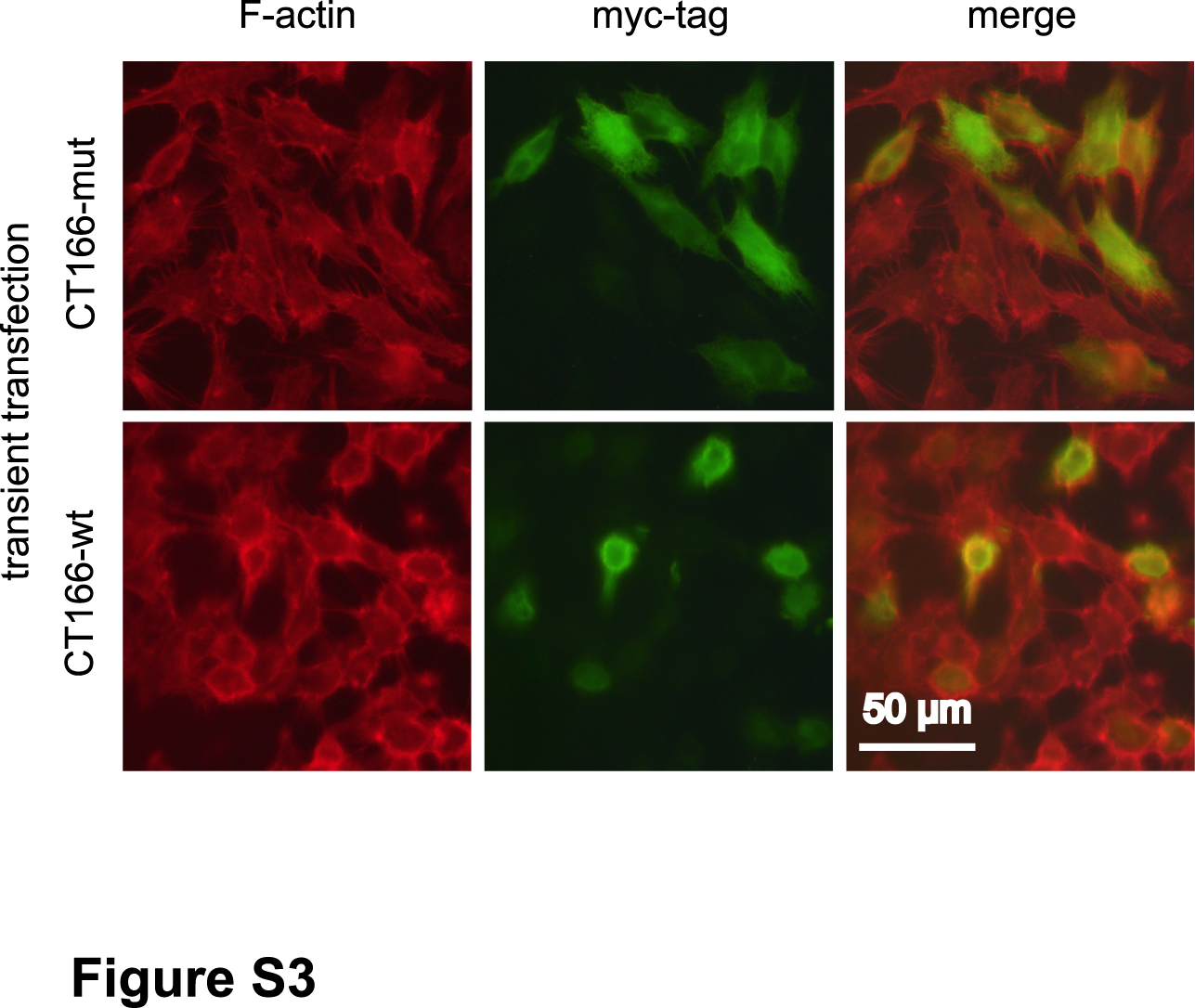

Supplement: Figure S3 — Cytopathic effect in HeLa cells transiently expressing CT166. HeLa cells were transiently transfected to express myc-tagged CT166-wt or CT166-mut. Expression of the recombinant proteins was determined by immunofluorescence using mouse anti-myc and anti-mouse IgG-FITC. Morphological changes of the actin cytoskeleton stained with rhodamine-phalloidin are already visible in low CT166 expressing HeLa cells. One representative experiment of three is depicted. (2.14 MB TIF) [file pone.0009887.s003.tif]

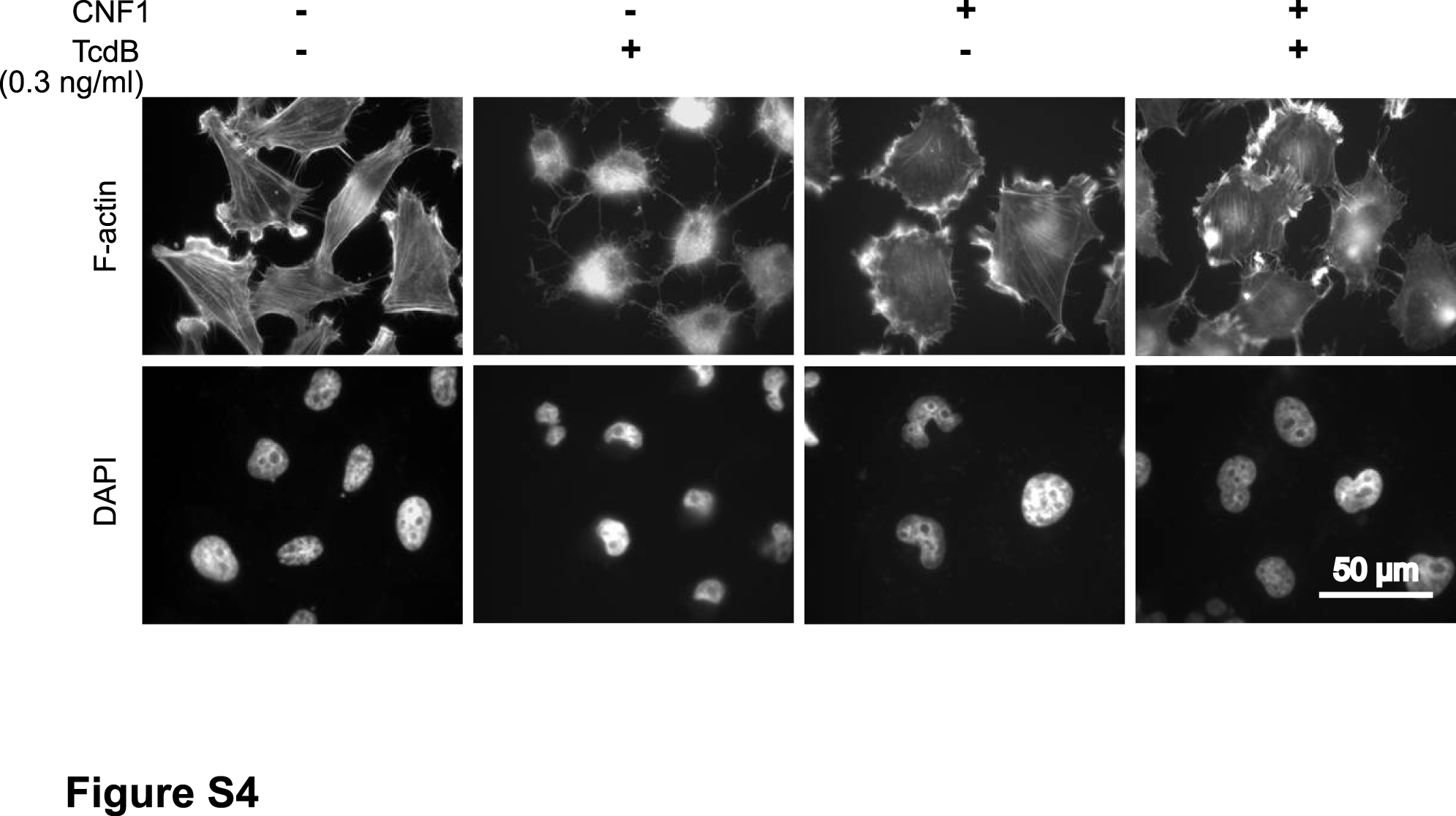

Supplement: Figure S4 — CNF1 prevents TcdB-induced morphological changes in HeLa cells. HeLa cells were incubated with 15 µg/ml CNF1 from E. coli for 6 h, or with 1 ng/ml TcdB for 2 h, or with CNF1 for 6 h followed by TcdB for another 2 h, as indicated. (1.28 MB TIF) [file pone.0009887.s004.tif]

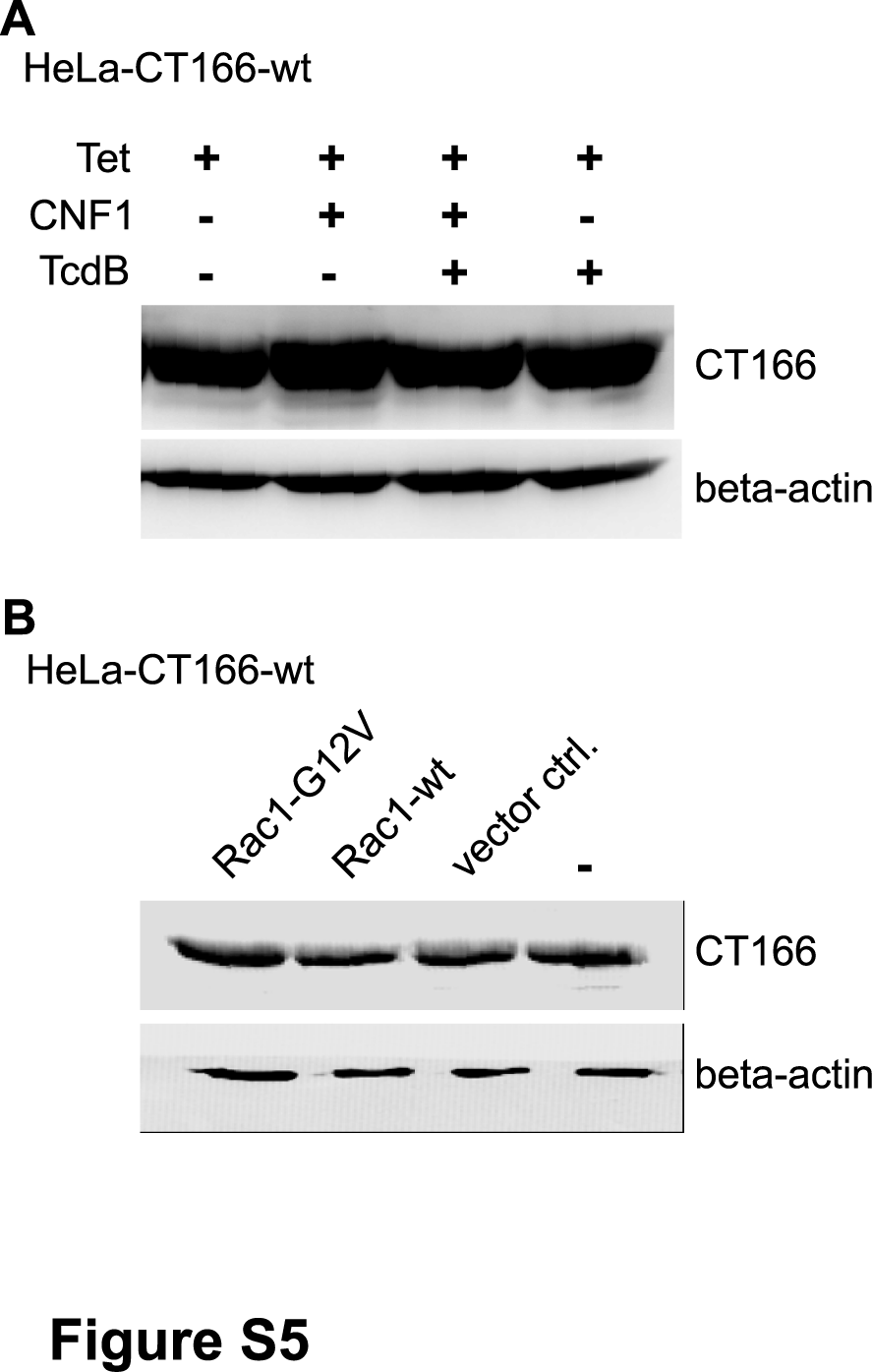

Supplement: Figure S5 — Neither CNF1 or TcdB treatment, nor Rac1 over-expression reduces CT166-wt expression. HeLa-CT166-wt cells were incubated with 1 µg/ml tetracycline for 24 h. Subsequently, cells were incubated with 15 µg/ml CNF1 from E. coli for 6 h, or with 1 ng/ml TcdB for 2 h, or with CNF1 for 6 h followed by TcdB for another 2 h, as indicated (A). HeLa-CT166-wt cells were transiently transfected with the pEGFP-C1 vector encoding for human Rac1-G12V or Rac1-wt, respectively, or the empty control vector following incubation with 1 µg/ml tetracycline for 24 h. Another 24 h after transfection, lysates were prepared (B). Lysates were analyzed for CT166 expression by Western blot, using anti-CT166 antiserum. Beta-actin was determined for a loading control. (0.26 MB TIF) [file pone.0009887.s005.tif]

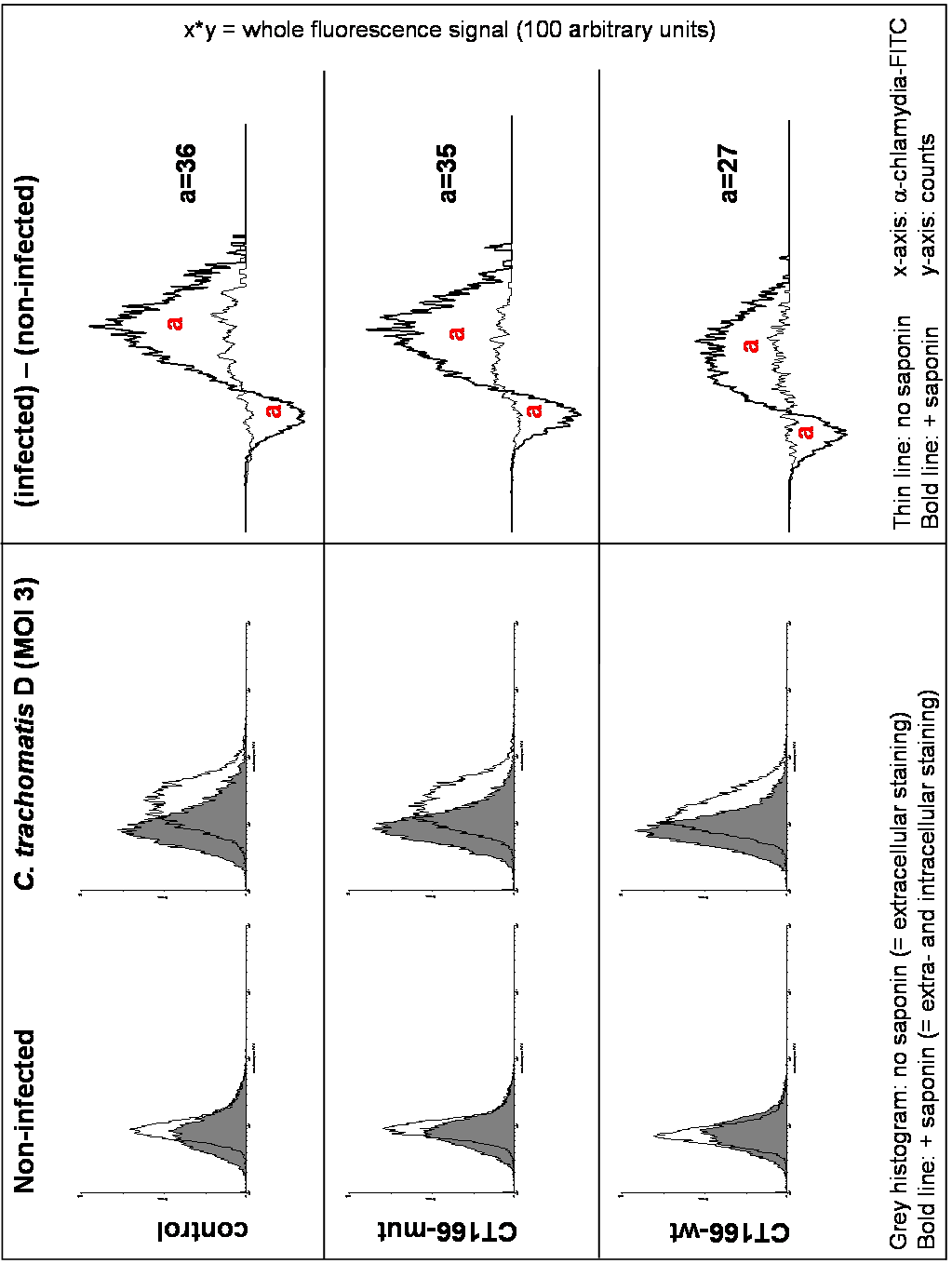

Supplement: Figure S6 — Uptake of C. trachomatis serovar D is impaired by CT166 expression - flow cytometry histogram supporting Figure 10A. HeLa clones were infected with C. trachomatis serovar D (MOI 3). Chlamydial uptake by HeLa-CT166-wt, HeLa-CT166-mut, or HeLa-control cells was determined by flow cytometry at 2 h p.i.. The x-axis of the depicted histograms presents the intensity of the anti-chlamydia-FITC signal; the y-axis presents the number of counts; grey histogram: without saponin (extracellular staining); bold line: with saponin (extra- and intracellular staining). The right panel (expressed as arbitrary units) is showing the fluorescence exclusively from intracellular chlamydiae. The calculation method allows to exclude non-specific signals from the host-cell and from extracellular chlamydiae bound to the cells surface. Depicted is one experiment as an example for the calculation method. (0.11 MB TIF) [file pone.0009887.s006.tif]
